# Efficient Entanglement Swapping in Quantum Networks for Multi-User Scenarios †

**DOI:** 10.3390/e27060615

**Published:** 2025-06-09

**Authors:** Binjie He, Seng W. Loke, Luke Lu, Dong Zhang

**Affiliations:** 1 College of Computer and Data Science, Fuzhou University, Fuzhou 350108, China; 210310008@fzu.edu.cn; 2 School of Information Technology, Deakin University, Melbourne, VIC 3125, Australia; seng.loke@deakin.edu.au; 3 Cisco Systems Inc., Suzhou 215123, China; luklu@cisco.com; 4 Fujian Province Key Laboratory of Information Security of Network System, Fuzhou University, Fuzhou 350108, China; 5 Zhicheng College, Fuzhou University, Fuzhou 350002, China; 6 Quan Cheng Laboratory, Jinan 250014, China

**Keywords:** quantum networks, entanglement swapping, quantum communication

## Abstract

Entanglement swapping is a crucial step in quantum communication, generating long-distance entanglements between quantum users for quantum network applications, such as distributed quantum computing. This study focuses on the efficiency of entanglement swapping strategies in quantum networks, particularly in multi-user concurrent quantum communication. Since multi-user concurrent quantum communication consists of multiple point-to-point quantum communications, we first analyze the challenges faced by existing entanglement swapping strategies in this scenario and then propose Parallel Segment Entanglement Swapping (PSES) to address them. PSES utilizes a tree-like model to divide the path into segments and execute entanglement swapping in parallel across them, thereby enhancing the generation rate of long-distance entanglement. Furthermore, we analyze the impact of resource contention on entanglement swapping in multi-user concurrent quantum communication and propose Multi-user PSES (M-PSES) to alleviate this negative impact. M-PSES leverages the entanglement swapping trigger signal and resource locking mechanisms to mitigate resource contention. The simulation results show that PSES performs superiorly to existing entanglement swapping strategies in point-to-point quantum communication, and M-PSES can achieve better performance than PSES in multi-user concurrent quantum communication.

## 1. Introduction

In recent years, with the development of quantum information technology, quantum computing [1] has become a high-profile research topic. Quantum computing has the potential to efficiently solve large-scale and complex scientific problems (e.g., chemical simulations, financial models, and machine learning) [2]. However, due to environmental noise and material technology, a single quantum computer can not integrate qubits on a large scale, limiting its computing power [2]. Quantum networks [3] can connect multiple quantum computers to enable collaboration, making distributed quantum computing [4] possible. Researchers are currently working on the architecture [5,6,7] and protocols [8,9,10,11,12] of quantum networks, but they are still in their infancy. It is worth mentioning that high-dimensional quantum systems (i.e., qudits) generated by quantum frequency combs [13] may play an important role in future quantum networks, as they can carry more information than qubits. Qubits are highly susceptible to decoherence due to channel noise, preventing quantum networks from transmitting quantum information over long distances via optical fibers. Instead, quantum teleportation [14] is required for long-distance qubit transfer. The prerequisite for quantum teleportation is the existence of long-distance entanglements between quantum users. Entanglement swapping (ES) serves as a fundamental technique for establishing long-distance entanglements in quantum networks. ES refers to performing a Bell-state measurement (BSM) on two qubits from distinct entanglements, thereby projecting the remaining two qubits into an entangled state [15]. This paper focuses on the efficiency of ES, particularly in multi-user concurrent quantum communication scenarios. Since multi-user concurrent quantum communication consists of multiple point-to-point quantum communications, it is both necessary and important to initially investigate efficient ES strategies for point-to-point quantum communication. We first propose an innovative strategy called Parallel Segment Entanglement Swapping (PSES) to improve the efficiency of ES in point-to-point quantum communication. Then, we analyze the impact of resource contention on the efficiency of ES in multi-user concurrent quantum communication scenarios and propose Multi-user PSES (M-PSES) to mitigate this adverse effect.

We should note that this paper represents a significant extension of our previous work [16], introducing plenty of substantial new research contributions and conclusions, including, but not limited to, the following aspects:

- PSES is extended to M-PSES to mitigate resource contention in multi-user concurrent quantum communication and further improve ES efficiency (Section 6).
- We add simulation experiments of M-PSES to demonstrate and discuss its performance improvements in multi-user concurrent quantum communication (Section 7).
- We extend and supplement the content related to PSES, such as explaining the reason for choosing heuristic algorithms to implement PSES (Section 5.4.1), conducting algorithm-level experiments for PSES (Section 5.4.3), and justifying the principle of parallel ES in PSES (Section 5.5).
- We add necessary resources and additional analysis, such as providing preliminaries and background (Section 2), additional related work (Section 3), and additional analysis of existing ES strategies (Section 4).

**Figure 1:**
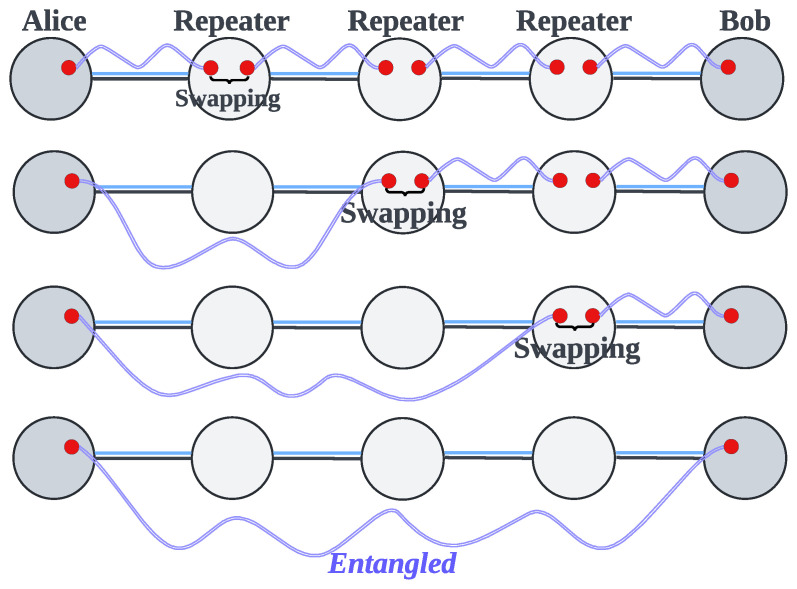
Sequential ES strategy. Repeaters perform ES one by one to generate long-distance entanglement between Alice and Bob. Because only one repeater can perform ES each time, the generation rate of long-distance entanglement is low.

In quantum networks, ES is performed in quantum repeaters. Sequential ES [17,18] serves as the simplest strategy for long-distance quantum communication. As shown in Figure 1, repeaters perform ES sequentially, executing one operation at a time. Generally, the entanglement fidelity, also known as the ratio of the Bell state when the entanglement involves a Werner state [19], affects the ES efficiency of repeaters. Under ideal conditions, entanglements are perfect Bell states (i.e., fidelity = 1), resulting in the highest and same ES efficiency across all repeaters. Under realistic conditions, entanglements deviate from perfect Bell states (i.e., 0 < fidelity < 1) because of environmental noise, which may result in different ES efficiencies among repeaters. Nevertheless, under either condition, the generation rate of long-distance entanglement in sequential ES remains relatively low because ES is performed by only one repeater at a time. To enhance the generation rate of long-distance entanglement and reduce the time overhead of ES, researchers have proposed parallel ES strategies, which are based on the core idea of allowing some nodes to perform ES simultaneously. Existing parallel ES strategies can be categorized into two types: the Balanced Binary Tree (BBT) strategy [20,21,22,23] and the Imbalanced Binary Tree (IBT) strategy [24]. The BBT strategy directly transforms the path into a balanced binary tree, where the nodes represent repeaters or users, allowing parent nodes on the same layer to perform ES simultaneously. The IBT strategy transforms the path into an imbalanced binary tree based on the ES efficiency of the nodes, thereby further reducing the time overhead of ES. However, our analysis reveals that both BBT and IBT face several challenges (see Section 4 for details), including suboptimal efficiency, the absence of a time synchronization mechanism, a lack of an effective ES failure processing mechanism, and unsuitability for multi-user concurrent quantum communication.

To overcome the limitations of existing ES strategies, we first introduce the PSES strategy for point-to-point quantum communication. PSES is fundamentally based on dividing the path into segments and adopting the mechanism of “performing parallel ES between segments and sequential ES within segments”. We further propose M-PSES to mitigate the resource contention problems in multi-user concurrent quantum communication. The core idea of M-PSES is to maximize parallel ES among nodes across different paths by setting trigger signals for ES execution and implementing resource locking control. We present our main contributions below.

(a) *Proposing PSES to improve ES efficiency in point-to-point quantum communication.* We utilize environmental noise (e.g., channel noise, depolarizing noise, and dephasing noise) to quantitatively evaluate the ES time cost at each node, which serves as the foundation of PSES. A tree-like model is proposed as the carrier of PSES. Furthermore, we present two algorithms, Layer Greedy and Segment Greedy, which segment nodes along the path based on node ES time costs and transform the path into a tree-like model. The simulation results show that PSES has a lower average ES time than existing strategies.

(b) *Implementing a time synchronization mechanism.* Leveraging the central controller of the hierarchical quantum network architecture [7], we design and implement a time synchronization control program to ensure the smooth execution of parallel ES.

(c) *Proposing an on-demand retransmission mechanism for ES failure.* We employ the central controller to promptly identify nodes where ES has failed and perform targeted re-preparation of entanglement. The simulation results demonstrate that the on-demand retransmission mechanism reduces both the time cost and entanglement consumption by approximately 80% in comparison to the traditional full-path retransmission approach.

(d) *Proposing M-PSES to enhance ES efficiency in multi-user concurrent quantum communication.* We design a resource locking mechanism and an ES trigger signal mechanism to mitigate resource contention, thereby maximizing parallel ES between nodes belonging to different paths. The simulation results indicate that M-PSES achieves a lower average ES time than PSES in multi-user concurrent quantum communication.

The rest of this paper is organized as follows. Section 2 introduces the preliminaries and background. Section 3 presents the related work. Section 4 analyzes and summarizes the challenges faced by existing ES strategies. In Section 5, we propose the PSES strategy. Section 6 discusses resource contention problems in multi-user concurrent quantum communication and proposes M-PSES. Section 7 shows the performance evaluation with simulation experiments. In Section 8, we conclude this work.

## 2. Preliminaries and Background

**Qubit.** The qubit is the basic unit of a quantum system [4]. Unlike the classical bit, which can only represent 1 or 0 at the moment, the qubit can represent not only 1 and 0 (denoted by |1〉 and |0〉), but also the superposition of 1 and 0 (denoted by $\alpha_{0}|0\rangle +\alpha_{1}|1\rangle$). $\alpha_{0}$ and $\alpha_{1}$ are amplitudes represented by complex numbers, and have ${\left|\alpha_{0}\right|}^{2}+{\left|\alpha_{1}\right|}^{2}=1$. The primary purpose of quantum communication is to transfer the qubit of a specific quantum state between two quantum systems.

**Entanglement.** Quantum entanglement [15] refers to the entanglement of multiple qubits through preparation or interaction so that the quantum state of each qubit cannot be described independently of the other qubits. Entanglements have the feature of nonlocality; once the state of one qubit in the entanglement has been changed, it will immediately affect the state of the other qubits, no matter how far apart they are.

**Entanglement swapping.** In quantum communication, ES [15] refers to generating long-distance entanglement between two distant devices through appropriate measurement (i.e., BSM) of short-distance entanglements and classical information assistance. ES is an important part of quantum communication and is used to overcome channel noise for long-distance quantum communication.

**Figure 2:**
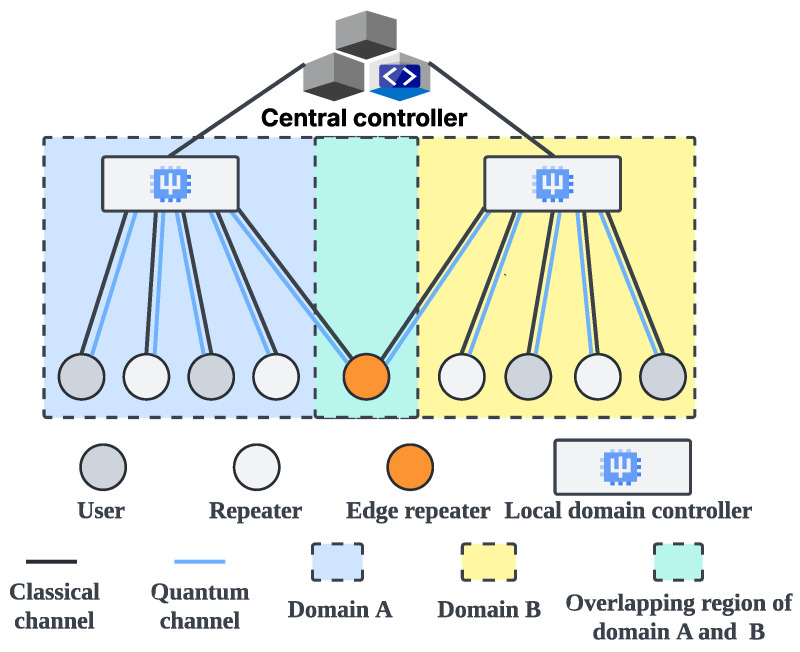
Hierarchical quantum network architecture. The central controller collects comprehensive information (e.g., environmental noise, network topology, and device status) from the entire quantum network via classical channels, while sending operational instructions to quantum devices. The local domain controller can prepare and distribute entanglement for domain quantum devices. In the hierarchical architecture, long-distance quantum communication refers to inter-domain quantum communication, which requires entanglement swapping on edge repeaters to generate long-distance entanglement.

**Quantum network architecture.** Quantum network architecture is an important component of research on the construction of quantum networks. The distributed quantum network architecture [5,6] is flat; each device has an independent control plane, and the quantum network uses repeaters for entanglement preparation and distribution. The hierarchical quantum network architecture [7] is a three-layer structure with a unified control plane, as shown in Figure 2. The central controller is responsible for the information collection and network control, and the local domain controller is responsible for the entanglement preparation and distribution. Because the central controller in the hierarchical architecture can control all quantum devices and collect their information, it is beneficial to realize time synchronization and effective ES failure processing mechanisms in parallel ES.

**Figure 3:**
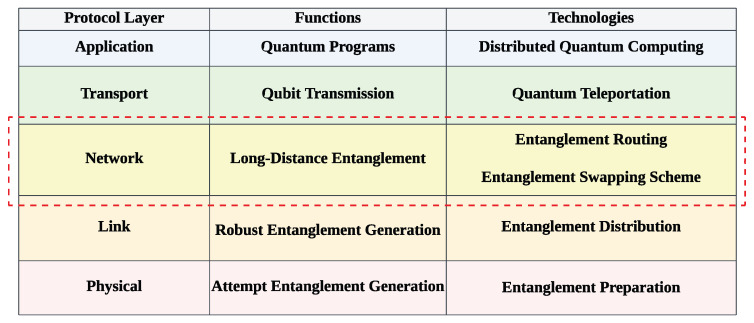
Quantum network protocol stack. The study of entanglement swapping strategies pertains to the network layer, as shown in the red dotted box.

**Quantum network protocol stack.** Similarly to classical networks, the protocol stack of quantum networks is divided into the physical, link, network, transport, and application layers [3], as shown in Figure 3. The ES strategies proposed in this paper operate at the network layer. In the network layer, the function of entanglement routing is to select an end-to-end path, and this study aims to apply a suitable ES strategy to the selected path.

**Environmental noise.** Unlike classical information, quantum information is susceptible to environmental noise. The following parameters can express environmental noise [17]:

- The depolarizing rate represents the probability that the qubit in quantum memory will depolarize with time.
- The dephasing rate represents the probability that the qubit will dephase with time.
- The Q-channel loss init rate represents the initial probability of a photon being lost upon entry to a quantum channel.
- The Q-channel loss noise represents the noise of the quantum channel, and the unit is dB/km.

## 3. Related Work

Neil et al. [25] studied the generation rate of long-distance entanglement with the influence of environmental noise, and pointed out that ES should be completed as soon as possible, because long-distance entanglement generation may fail due to prolonged time. Dai and Towsley [26] proposed that when performing ES in a quantum communication path, a node operation is likely to depend on the ES result of another node at a distance. The above two studies provide two basic facts: (a) the ES strategy should focus on enhancing efficiency, since long-distance quantum communication is highly sensitive to time; (b) the design of the ES strategy should carefully account for the dependencies between nodes.

Sequential ES [17,18] is the most straightforward strategy, where nodes on the path perform ES one by one. However, Shchukin et al. [27] highlighted that in multi-hop quantum communication, a post-order node must wait for the completion of ES by the preceding nodes before proceeding with its own operations. The waiting time is primarily influenced by the ES success rate of the preceding nodes, as ES failures require additional time for retries. The above research implies that long-distance quantum communication is likely to encounter failure in a noisy environment due to the prolonged waiting times of sequential ES.

Compared to sequential ES, parallel ES can significantly reduce waiting times and improve efficiency. However, its shortcoming is that it requires more complex control to ensure the smooth execution of parallel operations. The Balanced Binary Tree strategy [20,21,22,23] is a class of parallel ES. The core idea of the BBT strategy is to directly transform the path into a balanced binary tree, where the parent nodes perform ES to generate new entanglement between the child nodes. Parent nodes at the same layers can perform ES in parallel. The BDCZ [20], DLZC [21,22], and RED [23] are three representative studies of the BBT strategy. The BDCZ [20] partitions the path into multiple segments of equal length, with ES and entanglement purification carried out concurrently between the segments to maintain the fidelity of long-distance entanglement at an acceptable level. The DLZC [21,22] employs atomic ensembles and linear optics to realize the fundamental concepts of the BDCZ protocol. The RED [23] uses mathematical tools to analyze the process of forming the optimal balanced binary tree under ideal environmental noise conditions. Although the BBT strategy significantly improves the efficiency of ES, it does not consider the environmental noise differences between nodes when forming the balanced binary tree. These differences can result in varying ES time costs between nodes. When the differences in ES time costs between nodes become sufficiently large, idle waiting time among parallel nodes at the same layer may increase, thereby reducing efficiency. The Imbalanced Binary Tree strategy [24] is another class of parallel ES. The fundamental concept of the IBT strategy is to map the communication path into an imbalanced binary tree according to the ES efficiency of nodes. IBT aims to align the ES time costs of parent nodes at the same layer, thereby minimizing idle waiting time and enhancing efficiency. However, the ES efficiency of nodes is subject to fluctuations due to time-varying environmental noise [28,29], such as channel noise, dephasing noise, and depolarizing noise. These dynamic factors introduce randomness and volatility, making it challenging for IBT to find parallel nodes with similar ES time costs.

We should also mention the study of quantum network architecture, because it influences the implementation and design of ES strategies. The distributed architecture [5,6] usually includes users, repeaters, classical channels, and quantum channels, which means that ES strategies can only be deployed on distributed devices, relying on local information to perform ES. The hierarchical architecture [7] includes the central controller, local domain controllers, users, repeaters, classical channels, and quantum channels. The central controller is a unified control plane that collects environmental noise information across the entire network and controls the process of quantum communication. Therefore, ES strategies can be deployed in the central controller, leveraging its capabilities to improve device cooperation (e.g., time synchronization control and ES failure processing) during parallel ES.

## 4. Analysis of Existing Entanglement Swapping Strategies

Compared to the sequential ES strategy, parallel ES strategies can reduce the ES time cost and increase efficiency. However, both sequential and parallel ES strategies face challenges and problems, which we analyze in this section. To enhance clarity, we provide a brief definition of the node ES time cost, which is elaborated in Section 5.2. Node ES time cost refers to the time duration required for a node to complete ES successfully. Additionally, we need to clarify that a successful ES in a node refers to the generation of a new valid entanglement with a fidelity greater than 0.5 [30]. Due to environmental noise, failure is probable in the ES, which means repeated attempts may be necessary [15]. Each retry requires additional time for entanglement preparation, distribution, and swapping. Therefore, the higher the node environmental noise, the greater the probability of ES failure, the more retries are needed, and consequently, the higher the node ES time cost.

### 4.1. Suboptimal Efficiency

**Figure 4:**
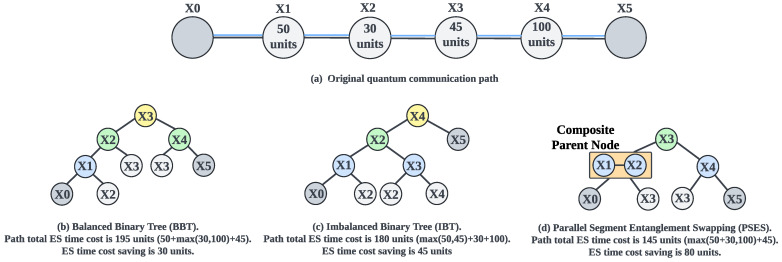
Examples of different ES strategies. Parent nodes of the same color perform parallel ES to generate entanglements between their leftmost and rightmost leaf nodes. Blue, green, and yellow represent the first, second, and third layers (rounds) of parallel ES, respectively. The layer ES time cost only equals the maximum time cost of the parent node, thereby saving the time cost of the remaining parent nodes. The path total ES time cost is the sum of the time cost of all the layers. (**a**) The original path is composed of users and repeaters. Each repeater has a different ES time cost, which is labeled inside the icon. (**b**) BBT transforms the path into a balanced binary tree to enable parallel ES. (**c**) IBT transforms the path into an imbalanced binary tree based on the node ES time cost. (**d**) PSES segments the path and forms a tree-like model. The repeaters inside the composite parent node perform sequential ES.

The sequential ES strategy [17,18] forces repeaters to perform ES one after another, preventing time savings and resulting in low efficiency. Suppose we have a path consisting of six nodes, as illustrated in Figure 4a. When applying sequential ES along this path, the total ES time cost is calculated by summing the ES time costs of all individual repeaters. In this example, the total ES time cost of the path is 50 + 30 + 45 + 100 = 225 units.

BBT [20,21,22,23] and IBT [24] are two types of parallel ES strategies that can reduce time costs and improve efficiency compared to the sequential approach. However, due to the shortcomings of their binary tree model, they still suffer from suboptimal efficiency, which we will analyze through the concrete examples shown in Figure 4. Figure 4 is not a special case, as it reflects the fundamental principles of different strategies. Changes in path length and ES time costs only enlarge the tree model, without affecting the conclusions of our analysis.

The main idea of BBT [20,21,22,23] is to convert the path into a balanced binary tree and parallelly perform ES at the parent nodes that belong to the same layer to generate new entanglement between the child nodes. In our example, BBT transforms the path shown in Figure 4a into the balanced binary tree shown in Figure 4b. During parallel ES, the first round performs ES at the parent node x1 in the first layer to generate a new entanglement between the child nodes x0 and x2. The second round performs ES in parallel at the parent nodes x2 and x4, generating entanglement between the child nodes x0 and x3, as well as x3 and x5, respectively. Subsequent layers proceed similarly, and ultimately, we obtain a long-distance entanglement between users x0 and x5. We should also mention that although it is not physically forbidden to perform ES simultaneously at all repeaters, doing so would significantly increase the ES failure rate and reduce the generation rate of long-distance entanglement (see Section 5.5 for a detailed explanation). Therefore, parallel ES strategies need the binary tree model to control the number of repeaters performing parallel ES in each round. In this case, we can calculate the total ES time cost for BBT. As shown in Figure 4a, the time cost for the first layer is 50 units; the time cost for the second layer is the maximum time cost of nodes x2 and x4, that is, max(30,100)=100 units, as these two nodes perform ES in parallel; and the time cost for the third layer is 45 units. Thus, the total ES time cost for the path is 50+max(30,100)+45=195 units. Compared to the sequential ES strategy, BBT saves 30 units of ES time cost. However, a limitation of BBT is that it prioritizes balancing the tree structure without considering the ES time cost of each node, which constrains its overall efficiency. This shortcoming becomes observable when comparing BBT with the other strategies in Figure 4.

The fundamental concept of the IBT [24] is to convert the path into an imbalanced binary tree based on the ES time cost of each node to ensure that the ES time costs of parent nodes at each layer are as closely matched as possible, thereby saving more time and optimizing efficiency. In our example, IBT transforms the path shown in Figure 4a into the imbalanced binary tree shown in Figure 4c. The parallel ES execution method is the same for both IBT and BBT, so we will not repeat it here. From Figure 4c, we can calculate the total ES time cost for IBT, which is max(50,45)+30+100=180 units. Compared to the sequential ES strategy, IBT saves 45 units of ES time cost, which is more efficient than BBT. However, there can be considerable differences in the ES time costs between nodes due to potentially significant differences in environmental noise between nodes. This makes it difficult for IBT to find nodes with similar ES time costs for forming an optimal imbalanced binary tree, thereby limiting its efficiency. In fact, the disadvantage of IBT is that it limits the parent node to consisting of only a single repeater, which makes its efficiency less ideal in reality than in theory. A deeper analysis of the underlying cause reveals that the primary issue stems from the binary tree model, which confines each parent node to a single repeater. This restriction results in a lack of flexibility in adjusting the ES time cost of the parent node. This shortcoming becomes apparent when comparing IBT with our proposed PSES shown in Figure 4d.

PSES divides the path into segments and generates a tree-like model, allowing composite parent nodes to exist. A composite parent node is an abstract parent node composed of multiple repeaters. The composite parent node and its child nodes are called a segment. In the tree-like model, the ES execution principle is “performing parallel ES between segments and sequential ES within segments”. We present the design and implementation of PSES in Section 5. The advantage of PSES lies in introducing the tree-like model, which provides adjustable flexibility in the ES time cost of the parent node. In other words, by combining different repeaters to form composite parent nodes, we can adjust the ES time cost to some extent, thereby increasing the probability of achieving similar ES time costs between parent nodes at the same layer. As illustrated in Figure 4d, nodes x1 and x2 form the composite parent node in the first layer, and they perform sequential ES with an ES time cost of 50 + 30 = 80 units. Meanwhile, the other parent node x4 in the same layer performs parallel ES with the composite parent node. Then, node x3 performs ES to generate long-distance entanglement between users x0 and x5. As a result, the total ES time cost for the path is max((50+30),100)+45=145 units. Compared to the sequential ES strategy, PSES saves 80 units of ES time cost, which is more efficient than IBT and BBT.

In conclusion, although Figure 4 is a simple example, it generally demonstrates that the suboptimal efficiency of IBT and BBT mainly stems from the binary tree model. Generally speaking, the greater the differences in ES time costs between nodes, the more difficult it becomes for the binary tree model to match nodes with similar costs, leading to poorer efficiency of IBT and BBT. On the contrary, the tree-like model introduced by PSES overcomes the limitations of the binary tree model, thereby effectively improving the efficiency of ES.

### 4.2. Time Synchronization

Parallel ES strategies involve multiple rounds of ES, where each round depends on the results of the previous one, thus requiring time synchronization to coordinate their execution. For example, in Figure 4c, the operation of node x2 in the second layer relies on the entanglements between x0 and x2, as well as x2 and x4, which are generated by the ES of nodes x1 and x3 in the first layer. Without time synchronization control, if node x2 starts its execution too early, the communication across the entire path will fail. However, the existing literature [20,21,22,23,24] does not discuss the issue of time synchronization.

A potential approach to addressing the time synchronization challenge involves equipping the quantum network with a unified control plane for time synchronization management. The central controller proposed in the hierarchical architecture [7] offers such a unified control plane, thereby establishing the foundational conditions required to tackle the time synchronization issue. Leveraging the strengths of the hierarchical architecture, we design and implement a time synchronization control program to support parallel ES (see Section 5.6).

### 4.3. Entanglement Swapping Failure

Because current quantum technologies are not yet capable of fully protecting qubits and entanglements from the effects of noise, which leads to reduced fidelity, ES failures may occur frequently in quantum communication. During the process of generating long-distance entanglement, if ES failures are not correctly handled, they can directly result in the failure of quantum communication. For example, in Figure 4c, if the ES at node x1 in the first layer fails, the entanglement between nodes x0 and x2 cannot be established. As a result, node x2 in the second layer cannot proceed with its operations, since it depends on the outcome of the first layer. Therefore, addressing ES failure is a critical challenge.

The approach to handling node ES failures in existing research [20,21,22,23,24] is the full-path retransmission mechanism. This mechanism refers to re-preparing short-distance entanglements across the entire path and retrying parallel ES. The reason why existing studies adopt the full-path retransmission mechanism lies in its simplicity, as it does not need to identify specific nodes where ES has failed, and instead re-prepares entanglements across the entire path. However, the mechanism introduces significant delays in long-distance quantum communication. As the number of nodes along the path increases, the probability of ES failures also rises, leading to a higher frequency of full-path retransmissions and greater time consumption. To address the drawback of the full-path retransmission mechanism, we propose a more efficient on-demand retransmission mechanism to process ES failures (see Section 5.6).

### 4.4. Unsuitability for Multi-User Concurrent Quantum Communication

Analogous to classical networks, future quantum networks are also expected to support concurrent communications among multiple users. However, unlike classical networks, quantum networks rely on quantum memories and channels to distribute and store entanglements to enable ES and generate long-distance entanglement. Due to the limited resources of quantum memories and channels, resource contention is likely to occur during multi-user concurrent quantum communication. For instance, when two paths intersect, they compete for the quantum memories at their common nodes. If the common nodes do not have sufficient quantum memories to serve both requests simultaneously, the two paths cannot proceed with communication in parallel. In addition, these paths may also contend for the quantum channels associated with the common nodes. Since multiple photons in the quantum channels must be transmitted with sufficient time intervals to avoid mutual interference, this further hinders multi-user concurrent quantum communication. A detailed analysis of the impact of resource contention on the efficiency of ES strategies is provided in Section 6.1.

Unfortunately, existing studies [20,21,22,23,24] do not address the issue of resource contention in multi-user concurrent quantum communication. In particular, both IBT and BBT are designed solely for point-to-point quantum communication. As illustrated in Figure 4, both IBT and BBT are limited to transforming a single path into a binary tree and performing parallel ES among the nodes inside the single path. Therefore, neither IBT nor BBT is suitable for multi-user concurrent quantum communication, as they do not consider the impact of resource contention on the efficiency of ES.

Nonetheless, research on ES strategies for point-to-point quantum communication is necessary and meaningful for multi-user scenarios, as multi-user concurrent quantum communication consists of multiple point-to-point quantum communications. For this reason, we first propose PSES for the point-to-point scenario and then extend it to the multi-user scenario by considering resource contention, leading to the design of M-PSES. We introduce the ES trigger signal and resource locking mechanisms for M-PSES, thereby enabling it to effectively mitigate the adverse effects of resource contention in multi-user concurrent quantum communication (see Section 6.2 and Section 6.3).

## 5. The Design of Parallel Segment Entanglement Swapping

### 5.1. Overview

The primary purpose of Parallel Segment Entanglement Swapping (PSES) is to enhance the ES efficiency of the optimal path selected by entanglement routing (e.g., Centralized Entanglement Routing [7]) in point-to-point quantum communication. We note that the different paths selected by various entanglement routing algorithms can affect the ES time cost. For example, if the routing algorithm selects a path that is idle but suffers from high environmental noise, then regardless of which ES strategy is used, the ES efficiency along that path will generally be lower than that of a path with much lower environmental noise. However, PSES can guarantee an enhancement in ES efficiency for the selected path. In other words, if the entanglement routing algorithm selects the path with the lowest environmental noise, PSES can provide a relatively optimal ES solution under the given noise conditions of that path. Given the current state of research, optimal entanglement routing algorithms, such as Centralized Entanglement Routing [7], can select the path with the lowest noise through evaluation formulae, which serves as a crucial foundation for the effectiveness of PSES. The core content of PSES is summarized as follows.

**Quantitative evaluation of node ES time cost.** Since environmental noise (e.g., depolarizing noise, dephasing noise, and channel noise) is a critical factor influencing the ES time cost, we quantitatively evaluate the node ES time cost based on the environmental noise, which serves as a prerequisite for implementing PSES. Specifically, we define the node ES time cost, present its quantitative evaluation formula, and explain the rationale behind the definition and the formula (Section 5.2).

**Tree-like model design.** The tree-like model acts as a carrier of PSES (Section 5.3). We propose heuristic algorithms, Layer Greedy and Segment Greedy, to transform a path into a tree-like model based on node ES time cost (Section 5.4).

**Principle of parallel entanglement swapping.** The principle of parallel ES in the tree-like model is “performing parallel ES between segments and sequential ES within segments”. A composite parent node and its children nodes are called a segment. Repeaters inside the composite parent node perform sequential ES, while parallel ES is performed between different parent nodes in the same layer (Section 5.5).

**Entanglement swapping process control.** We design a process control program deployed in the central controller of the hierarchical architecture [7] to perform time synchronization and deal with node ES failure (Section 5.6).

We outline the characteristics of PSES here. PSES is designed for point-to-point quantum communication, aiming to enhance the parallelism of ES among nodes within a single path. More importantly, PSES lays the foundation for efficient ES strategies in multi-user concurrent quantum communication, as improving the ES efficiency within a single path is a necessary step before extension to scenarios involving concurrent communication over multiple paths. PSES offers two main advantages. First, the tree-like model and the principle of “performing parallel ES between segments and sequential ES within segments” enable more nodes to complete ES simultaneously, thereby reducing the total ES time cost in the path and enhancing the efficiency of ES for point-to-point quantum communication. Second, in ES process control, the time synchronization and on-demand retransmission mechanisms ensure the reliability of the parallel ES and further reduce entanglement resource consumption.

### 5.2. Time Cost of Node Entanglement Swapping

We provide a detailed definition of the node ES time cost here. The node ES time cost is defined as the time required to complete successful ES at a node. Successful ES refers to ES that results in a new valid entanglement with fidelity greater than 0.5 [30]. However, environmental noise can affect the success rate of ES, leading to the possibility of failure [15]. Once an ES failure occurs, the node must retry until a new valid entanglement is generated. The time spent on these retries is also considered part of the node ES time cost. For instance, if a node attempts ES five times before achieving success, the cumulative time taken is referred to as the node ES time cost. Obviously, without considering other factors, nodes with higher environmental noise will require more retries compared to nodes with lower environmental noise, resulting in a higher node ES time cost. However, since ES failure is inherently a probabilistic event and influenced by physical systems (e.g., trapped ions or superconducting circuits) [31,32], we cannot precisely give the exact time consumed, such as 1 ms or 2 ms. Therefore, the node ES time cost represents a relative time length rather than an exact time. For example, we can compare that 2 units of node ES time cost takes longer than 1 unit, but we cannot specify that 2 units correspond to an exact time, such as 2 ms or 2 s.

We discuss how to quantitatively evaluate the node ES time cost based on environmental noise. It is important to note that because this research primarily focuses on the impact of environmental noise on ES, we assume that quantum networks have a constant entanglement preparation rate and two quantum memories at each repeater (i.e., only one ES can be executed at a time). The entanglement preparation rate and the number of quantum memories may impact ES, and are worth studying in the future. According to research [17], the dephasing rate is one of the ways to represent dephasing noise, and it can affect the quantum gate operation. The depolarizing rate is one of the ways to represent depolarizing noise, and it can affect the quantum memory efficiency. Channel noise can affect the quality of the Q-channel, thereby impacting the efficiency of short-distance entanglement distribution. Q-channel quality can be represented by the Q-channel loss init rate and Q-channel loss noise. Performing successful ES requires meeting the following three conditions:

**Condition A.** Short-distance entanglement distribution between nodes is successful; this is determined by the Q-channel quality [7,17].

**Condition B.** Short-distance entanglement is successfully stored in quantum memory; this is determined by the depolarizing rate [7,17].

**Condition C.** The quantum gate operations of ES succeed; this is determined by the dephasing rate [7,17].

We should note that a complete ES process in a broad sense involves entanglement distribution, qubit storage, and quantum gate operations. Therefore, from this perspective, we can state in plain terms that depolarizing noise, dephasing noise, and channel noise collectively impact ES. However, for the sake of clarity, we follow the narrow-sense perspective by decomposing ES into the above three steps and associating each type of noise with the corresponding step. Our strict compliance with this mapping is also evident in the simulation source code [33]. We propose Formula (1) to quantitatively evaluate the node ES time cost.

(1) $$NC=\frac{1}{CQ\times (1-DPZR)\times (1-DPSR)}$$

NC denotes the node ES time cost, while DPZR, DPSR, and CQ represent the depolarizing rate, dephasing rate, and Q-channel quality, respectively. Formula (1) is capable of representing the node ES time cost because CQ represents the probability of successful short-distance entanglement distribution, which is the probability of satisfying Condition A. CQ can be calculated using Formula (2), which will be explained later. (1 - DPZR) represents the probability of storing short-distance entanglement in quantum memory without depolarization, which is the probability of satisfying Condition B. (1-DPSR) represents the probability of successful ES operation, which is the probability of satisfying Condition C. Therefore, the denominator in Formula (1) indicates the probability that the repeater will successfully complete one ES attempt. As a result, its reciprocal represents the number of attempts the repeater needs to achieve successful ES, which corresponds to the node ES time cost. We provide an example to help illustrate Formula (1). In a perfect quantum network with no environmental noise, the depolarizing rate and dephasing rate are both 0, and the channel quality is 1, so the node ES time cost is 1, meaning one attempt is needed for ES to succeed. As environmental noise increases, additional attempts are necessary, resulting in an increase in the node ES time cost.

Based on study [7], the Q-channel quality is represented by Formula (2). We should note that the validity of Formula (2) is based on the assumption that the length of the quantum channel is 100 km, because when the noise of a 100 km channel is greater than or equal to 0.2 dB/km, short-distance entanglement distribution will experience 100% failure [7]. This assumption is practical for simulation verification because we can easily control the channel length in the simulator.

(2) $$CQ=1-\frac{QLIR+\frac{QLN}{0.2}}{2}$$

QLIR represents the Q-channel loss init rate, and QLN stands for Q-channel loss noise. Based on the above assumption, the values of the QLIR range from 0 to 1, while the QLN ranges from 0 dB/km to 0.2 dB/km. The correctness of Formula (2) has been proven in the literature [7]. We provide an example to help illustrate Formula (2). If a Q-channel is perfect, the QLIR is 0, and the QLN is also 0 dB/km, so the CQ equals 1, representing a 100% success rate for entanglement distribution. In the worst-case scenario, QLIR is 1, and QLN is 0.2 dB/km, so CQ equals 0, representing a 0% success rate for entanglement distribution.

### 5.3. Tree-like Model

The tree-like model serves as a carrier of PSES, reflecting how PSES performs parallel ES. It is called the tree-like model because it includes the composite parent node formed by multiple repeaters. Therefore, strictly speaking, the model is not a true tree. We use an example to illustrate the tree-like model. As shown in Figure 4d, the original path of Figure 4a is divided into two segments, {x0,x1,x2,x3} and {x3,x4,x5}, to form the first layer of the tree-like model. Each segment begins and ends with the leftmost and rightmost leaf nodes, respectively, while the intermediate nodes collectively form the composite parent node. The intermediate nodes are able to perform ES to create a new entanglement between the leftmost and rightmost leaf nodes. After the intermediate nodes in each segment of the first layer complete ES and generate new entanglements, all the leaf nodes form a new path, which can be further divided into segments to construct the second-layer structure of the tree-like model. For example, if ES succeeds at nodes x1, x2, and x4 in the first layer of Figure 4d, a new path, x0-x3-x5, is formed. This leads to the second-layer segmentation {x0, x3, x5}, where x3 serves as the parent node, x0 is the leftmost leaf node, and x5 is the rightmost leaf node. If the path is longer than that shown in Figure 4a, the tree-like model will have more layers.

We need to explain the execution mechanism of the tree-like model, which includes ES between layers, as well as among nodes within the same layer. Regarding the ES between layers, since the ES operation of the higher layer depends on the ES results of the lower layer, we must follow a bottom-up operation order, such as performing ES in the first layer and then proceeding to the second layer. Regarding the ES among nodes within the same layer, it follows the principle of “performing parallel ES between segments and sequential ES within segments”. For example, in the first layer of Figure 4d, two parent nodes, {x1, x2} and {x4}, which are part of the two segments {x0, x1, x2, x3} and {x3, x4, x5}, start performing ES simultaneously. However, within the composite parent node, the repeaters x1 and x2 should perform ES sequentially. In addition, Section 5.5 provides a detailed explanation of the necessity and rationale behind the principle of “performing parallel ES between segments and sequential ES within segments”.

PSES transforms a path into a tree-like model based on the node ES time cost, with the goal of ensuring the ES time costs among composite parent nodes at the same layer are similar, thereby minimizing the total ES time cost of the path. We provide the specific algorithms for generating the tree-like model in Section 5.4. Even though Figure 4 presents a simple case, the demonstrated advantages and characteristics of PSES are general. Compared with the traditional binary tree model in IBT and BBT, the main advantage of the tree-like model in PSES is the introduction of composite parent nodes, which can flexibly adjust the ES time costs of parent nodes by combining multiple repeaters, thereby reducing the idle waiting time during parallel ES. This allows more repeaters to perform ES in parallel.

### 5.4. Algorithms for Generating Tree-like Model

#### 5.4.1. Reasons for Choosing Heuristic Approach

**Figure 5:**
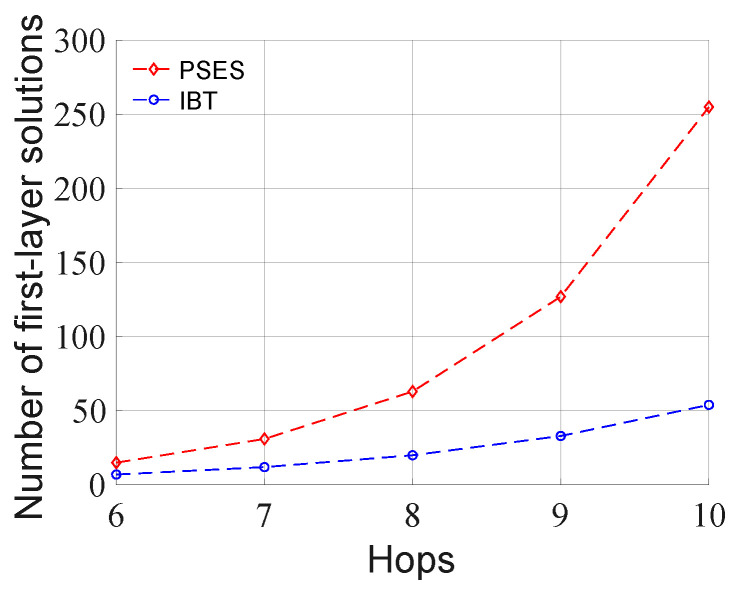
Possible first-layer solutions vs. hops.

There are two possible approaches to generating the tree-like model: an optimal approach (e.g., the Dynamic Programming algorithm) and a heuristic approach (e.g., the Greedy algorithm). Researchers have tried to use the Dynamic Programming algorithm to generate the binary tree model in IBT [24]. Although the Dynamic Programming algorithm can maximize the efficiency of parallel ES under the binary tree model, its time consumption is enormous due to the large number of traversal cases [24]. Figure 5 shows the number of possible solutions for the first layer in the tree-like model of PSES and the binary tree model of IBT at different path lengths. As the number of hops increases, the number of layer-level solutions in PSES grows faster than that in IBT. Thus, the number of tree-level solutions in PSES is much larger than that in IBT, indicating that the complexity of PSES is higher than that of IBT due to the introduction of composite parent nodes. Therefore, if we use Dynamic Programming for PSES, the number of traversal cases will be more than that of IBT, which means higher time consumption than that of IBT. For this reason, we do not choose the optimization approach instead of the heuristic one.

#### 5.4.2. Design of Heuristic Algorithms

We introduce two heuristic algorithms to transform a path into a tree-like model, namely Layer Greedy and Segment Greedy.

**Layer Greedy.** The core idea of Layer Greedy is to construct a tree-like model layer by layer. Layer Greedy treats the optimal layer solution as the greedy objective. Specifically, the layer solution that yields the greatest ES time cost savings is selected as the optimal solution. The pseudocode of Layer Greedy is presented in Algorithm 1. The inputs are the path and node ES time costs, and the output is a tree-like model. The Layer Greedy algorithm is divided into two steps, as shown below.

Step A: Layer Greedy finds the optimal solution for the first layer of the tree-like model. All the possible segment combinations in the original path are traversed. Specifically, as shown in lines 9 to 15 of Algorithm 1, we start by traversing layer solutions where the number of segments is one, and compute the corresponding time savings. This process continues until the number of segments reaches its maximum, ensuring all the possible layer solutions are traversed. For example, in the case of Figure 4a, {{x0, x1, x2, x3}, {x3, x4, x5}} and {{x0, x1, x2}, {x2, x3, x4, x5}} are two possible layer solutions when the number of segments is 2. Ultimately, the layer solution that achieves the greatest time savings is selected as the optimal solution for the first layer, as shown in lines 4 and 5 of Algorithm 1. After finding the first-layer solution, as indicated in lines 6 and 7 of Algorithm 1, the repeaters and users not selected as parent nodes form a new path, referred to as the remaining path, which is used for generating higher-layer solutions.

Step B: For the nth layer (n>1), Layer Greedy recursively performs step A on the remaining path of the (n − 1)th layer until the root, as shown in lines 1 to 8 of Algorithm 1.

**Segment Greedy.** The core idea of Segment Greedy is to construct a tree-like model segment by segment. Segment Greedy considers the optimal segment solution as the greedy objective. In Layer Greedy, we know that a layer solution consists of multiple segments, and because these segments can perform ES in parallel, the time savings can be calculated to select the optimal layer solution. However, in Segment Greedy, we cannot calculate the time savings for an individual segment. Therefore, Segment Greedy evaluates the benefit of forming a segment to select the optimal segment solution. In what follows, we discuss how to evaluate the benefit of forming a segment.
**Algorithm 1:** Layer Greedy.
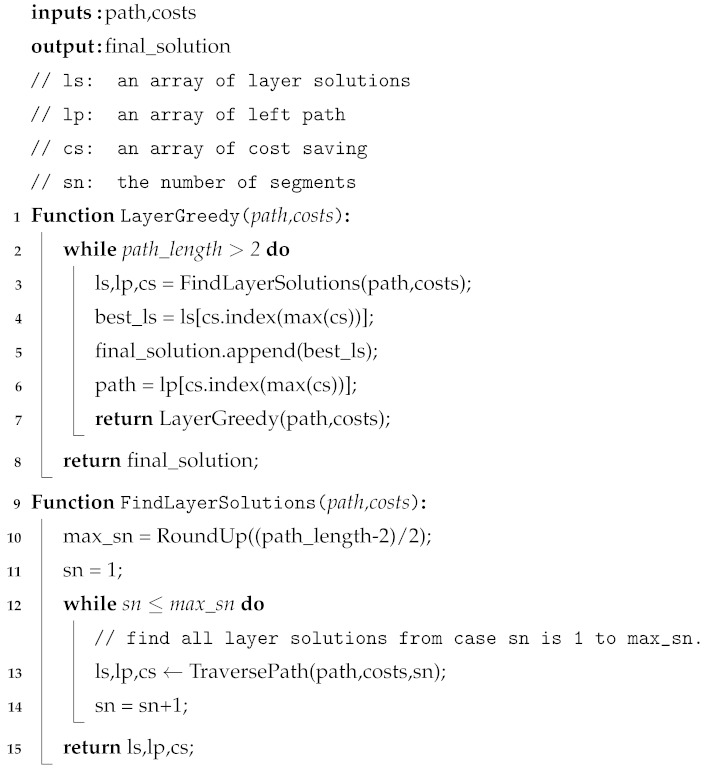


In PSES, segments can be categorized into two types: those with a single parent node (e.g., {x0, x1, x2}) and those with a composite parent node (e.g., {x0, x1, x2, x3}). Generally, we traverse each node to consider whether it can form a segment with its neighbors. For example, when we reach x2 during traversal, it can form a segment with a single parent node, such as {x1, x2, x3}. A segment with a composite parent node is formed by extending a segment with a single parent node. For example, adding x4 to {x1, x2, x3} obtains {x1, x2, x3, x4}.

We first discuss the benefit of forming the segment with a single parent node. The core principle for evaluating the benefit is to compare the ES time cost of the current segment with the maximum ES time cost among the existing segments in the current layer, as their difference reflects the additional time that can be saved by forming the current segment. When the ES time cost of the current segment is less than or equal to the maximum ES time cost among existing segments in the current layer, the additional time saving is equal to the ES time cost of the current segment, because of parallel ES having been performed between segments. In the above case, the net benefit is the ES time cost of the current segment. When the ES time cost of the current segment exceeds the maximum ES time cost, the additional time saving is equal to the maximum ES time cost. However, in the above case, because the maximum ES time cost among segments determines the total ES time cost of a layer, the negative aspect is that the time cost of the current layer also increases. Therefore, the net benefit should be calculated as the maximum ES time cost minus the increased time cost of the current layer. If the net benefit is positive, we temporarily treat the current segment as the optimal segment solution. We say “temporarily” because the segment with a single parent node can be extended into a segment with a composite parent node, which may offer the potential to further increase the net benefit. We propose Formula (3) to formally represent the above cases and evaluate the net benefit of forming the segment with a single parent node.

(3) $$\begin{array}{c} NBCS=\min(CCS,MSC)-\max(0,CCS-MSC) \end{array}$$

NBCS stands for the net benefit of the current segment, CCS for the ES time cost of the current segment, and MSC for the maximum ES time cost among existing segments in the current layer. In Formula (3), if CCS≤MSC, we have NBCS=CCS, which is the first case mentioned above. If CCS>MSC, we have NBCS=MSC−(CCS−MSC), which is the second case mentioned above. We can find that when the CCS is much greater than the MSC, the net benefit becomes a negative number.

We now discuss the benefit of forming a segment with a composite parent node. Because the segment with a composite parent node is extended from the segment with a single parent node, the core principle is evaluating the net benefit growth when forming the segment with a composite parent node. We refer to the currently computed segment with a composite parent node as the future segment to distinguish it from the segment with a single parent node. Formulas (4) and (5) are proposed to evaluate the net benefit growth when extending the segment with a single parent node into the segment with a composite parent node.

(4) $$\begin{array}{c} NBFS=\min(CCS+CN,MSC)-\max(0,CCS+CN-MSC) \end{array}$$

(5) NBG=NBFS−NBCS

NBFS stands for the net benefit of the future segment, CN for the ES time cost of the node, and NBG for the net benefit growth. The principle of Formula (4) is the same as that of Formula (3), so we do not repeat the explanation here. However, we note that CCS+CN represents the ES time cost of the segment with a composite parent node, as it is formed by adding a new node to the segment with a single parent node. Formula (5) shows the net benefit growth, which is the difference in net benefit between the future and current segments. If the net benefit growth is positive, we treat the future segment as the optimal segment solution.

The pseudocode of Segment Greedy is presented in Algorithm 2. Since a path of long-distance quantum communication consists of at least three nodes (i.e., two users and one repeater), Segment Greedy forms the first segment using the first three nodes by default, as shown in lines 3 and 4 of Algorithm 2. We then start traversing the path from the fourth node. As shown in lines 10 to 19 of Algorithm 2, if the traversed node is not a tail node of existing segments, we utilize Formula (3) to evaluate the net benefit to decide whether to form a new segment with a single parent node. As shown in lines 20 to 32 of Alg. 2, if the traversed node is a tail node of existing segments, we utilize Formulas (4) and (5) to evaluate the net benefit growth, in order to decide whether to add one more node to the current segment to form a new segment with a composite parent node. As shown in lines 33 to 34 of Algorithm 2, each iteration of the outermost loop in Segment Greedy produces a layer of the tree-like model, and then recursively invokes itself to generate the next layer until the root. In simple terms, Segment Greedy can be summarized in the following two steps.
**Algorithm 2:** Segment Greedy.
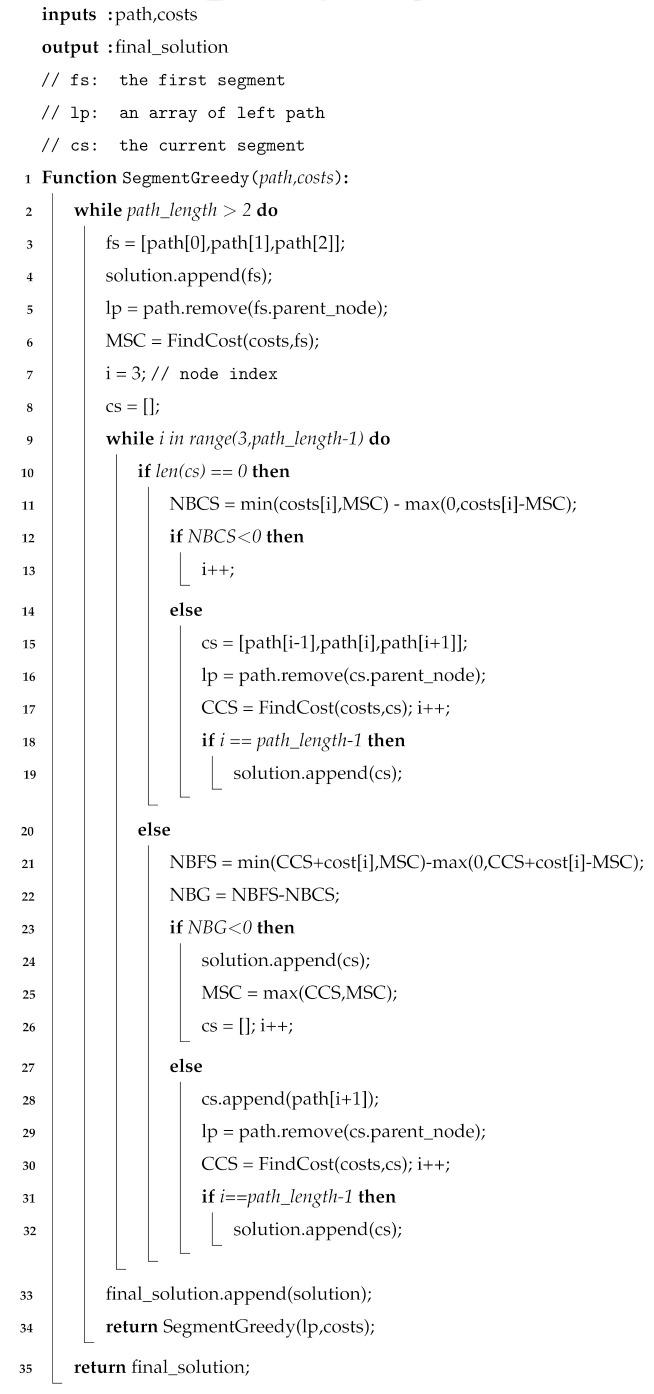


Step A: The original path is traversed one time, and a greedy decision is made on whether to form segments according to Formulas (3)–(5). After the traversal has been completed, the repeaters and users not selected as parent nodes form the remaining path.

Step B: Step A is recursively performed on the remaining path until the tree-like model has been generated.

#### 5.4.3. Assessment of Heuristic Algorithms

We summarize the difference between Layer Greedy and Segment Greedy in Table 1. Layer Greedy iteratively seeks the optimal layer solution instead of the whole tree, so its time complexity is lower than that of Dynamic Programming and higher than that of Segment Greedy. Besides, Layer Greedy may fall into the local optimum. Segment Greedy traverses the path only once at each layer to find the optimal segment solution, so it has the lowest time complexity, but may fall into the local optimum.

We performed experiments at the algorithm level to initially verify the performance of different algorithms implemented in different parallel ES strategies. The source code is published in [33]. We should emphasize that unlike the quantum network simulation experiment in Section 7, the advantage of the algorithm-level experiment is that it excludes the influence of environmental noise settings, allowing the focus to be solely on the performance of the algorithms. In the algorithm-level experiment, the core ideas of Layer Greedy and Segment Greedy were applied to IBT to make a fair comparison with PSES. We assumed that the node ES time cost had been quantified, and the original data of the quantified node ES time cost used in the experiment consisted of random numbers following a Gaussian distribution. We used the average ES time cost savings as a performance metric. Each data point in the results is the average of 100 repeated experiments.

**Figure 6:**
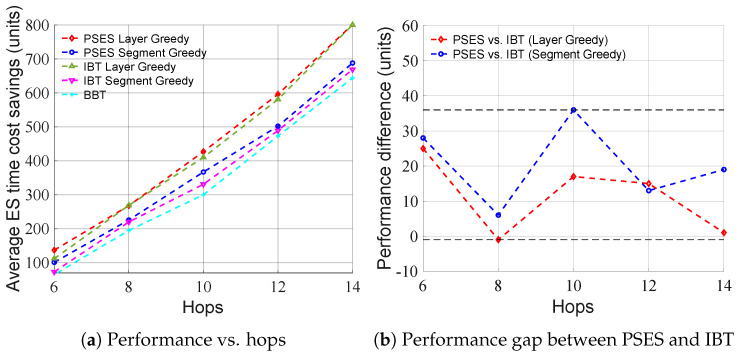
The average ES time cost savings of algorithms across different numbers of hops. In the algorithm-level experiments, the ES time cost of nodes along the path is randomly generated following a Gaussian distribution, where the average node ES time cost is fixed at 100 units and the standard deviation is fixed at 80 units. (**a**) Under different numbers of hops, the performance of the Layer Greedy consistently surpasses that of the Segment Greedy. (**b**) When the same algorithm (i.e., Layer Greedy or Segment Greedy) is adopted, increasing the number of hops does not expand the performance gap between PSES and IBT.

Figure 6 shows how performance changes as the number of hops increases under the condition that the average node ES time cost is 100 units and the standard deviation is 80 units. Figure 6a shows that the average ES time cost savings of all strategies rise as the number of hops increases, because the more hops there are, the more ES can be performed concurrently, and the more ES time costs can be saved. In addition, the performance order is PSES Layer Greedy > IBT Layer Greedy > PSES Segment Greedy > IBT Segment Greedy > BBT. Figure 6b shows that as the number of hops increases, the performance gap between PSES and IBT only fluctuates within a limited range, without continuously widening, indicating that longer paths do not further amplify the advantage of PSES. In conclusion, from the algorithmic perspective, the performance of Layer Greedy is always superior to that of Segment Greedy under different numbers of hops. From the perspective of parallel ES strategies, the PSES performance is always superior to that of IBT when adopting the same algorithm.

**Figure 7:**
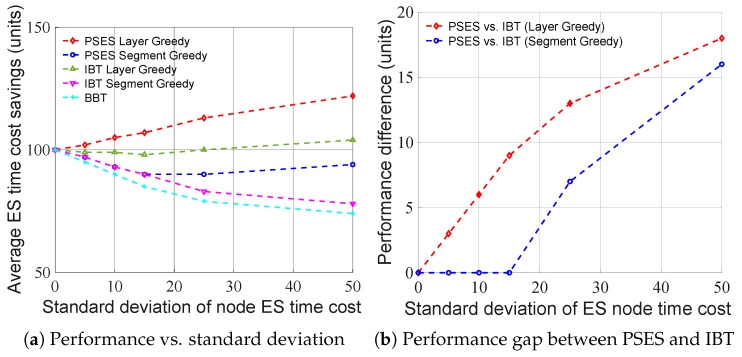
The average ES time cost savings of algorithms across different standard deviations. The ES time cost of nodes along the path is randomly generated following a Gaussian distribution, where the average node ES time cost is fixed at 100 units and the number of hops is fixed at 6. (**a**) Under different standard deviations, the performance of Layer Greedy consistently surpasses that of Segment Greedy. (**b**) When the same algorithm (i.e., Layer Greedy or Segment Greedy) is adopted, increasing the standard deviation widens the performance gap between PSES and IBT.

Figure 7 shows how the performance changes as the standard deviation of node ES time cost increases under the condition that the average node ES time cost is 100 units and the number of hops is 6. As can be seen from Figure 7a, the performance of Layer Greedy is always superior to that of Segment Greedy under different standard deviations. However, we can also observe that the performance of Segment Greedy may decrease as the standard deviation increases. This is because the problem size and granularity of Segment Greedy are smaller than those of Layer Greedy, making it more susceptible to getting trapped in the local optimum, leading to a performance decline. As can be seen from Figure 7b, as the standard deviation of the node ES time cost increases, the performance gap between PSES and IBT also increases. The reason for this is that PSES can form more composite parent nodes due to larger differences in node ES time costs, thereby saving more ES time costs than other strategies. In conclusion, from the algorithmic perspective, the performance of Layer Greedy is always superior to that of Segment Greedy under different standard deviations. From the perspective of parallel ES strategies, when adopting the same algorithm, the performance advantage of PSES relative to IBT grows as the standard deviation increases.

**Figure 8:**
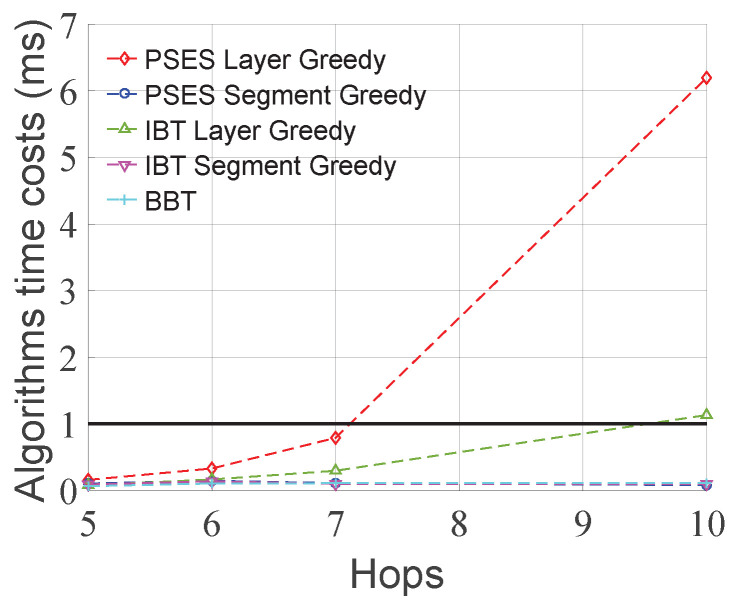
The time consumption of the different algorithms. The time consumption of Layer Greedy is higher than that of Segment Greedy. The time consumption of IBT Segment Greedy and PSES Segment Greedy is similar.

Figure 8 shows the time consumption of the different algorithms. It can be found that when the number of hops is greater than 7, the time consumption of PSES Layer Greedy and IBT Layer Greedy approaches milliseconds and has a significant upward trend. On the contrary, the time consumption of PSES Segment Greedy, IBT Segment Greedy, and BBT is similar, all at the microsecond level, with no apparent upward trend. In conclusion, the time consumption of Segment Greedy is much less than that of Layer Greedy.

Based on the above experimental results, we summarize the best-fit schemes for different scenarios in Table 2. In short-path scenarios (i.e., hops ≤ 7), the algorithm time cost of all schemes remains at the microsecond level. Therefore, we choose PSES Layer Greedy, which achieves the greatest ES time cost saving for short-path scenarios. In long-path scenarios (i.e., hops > 7), the algorithm time costs of both PSES Layer Greedy and IBT Layer Greedy increase with the number of hops and reach the millisecond level, while the other schemes still maintain time costs at the microsecond level. Hence, among the remaining schemes, we choose PSES Segment Greedy, which offers the best ES performance for long-path scenarios.

### 5.5. Principle of Performing Parallel Entanglement Swapping

In parallel ES, non-adjacent repeaters usually perform ES simultaneously to reduce time costs, thereby increasing the generation rate of long-distance entanglement [20,21,22,23,24]. However, this does not mean that performing parallel ES between adjacent repeaters is physically infeasible. In fact, once entanglement distribution has been completed for all repeaters, it is physically possible to perform ES on all repeaters simultaneously. The minimum ES time cost can be achieved if all repeaters complete ES successfully in a single attempt. However, due to the presence of environmental noise in the real world, it is nearly impossible for all repeaters to complete ES successfully in a single attempt. In this case, if all repeaters are allowed to perform ES simultaneously, the ES results of all repeaters are tightly interdependent at that exact moment. If even one repeater fails in ES, all repeaters must retry entanglement distribution and ES, which increases the time overhead. Therefore, in parallel ES, adjacent repeaters are usually restricted from performing ES simultaneously to reduce mutual dependencies.

In PSES, when executing the tree-like model, the principle of “performing parallel ES between segments and sequential ES within segments” is applied. “Performing parallel ES between segments” is easy to understand because the intermediate nodes of different segments (i.e., composite parent nodes) are non-adjacent, and performing parallel ES between them will not cause dependency issues. For example, in the two segments {x0, x1, x2, x3} and {x3, x4, x5} shown in Figure 4d, x1 and x4 are non-adjacent, as are x2 and x4. The reason for adopting “sequential ES within segments” is that intermediate nodes within the same segment are necessarily adjacent. For example, in the segment {x0, x1, x2, x3}, x1 and x2 are adjacent nodes. If x1 and x2 were to perform ES simultaneously, a dependency issue would arise, meaning that if either x1 or x2 fails in ES, the entire segment would need to retry entanglement distribution and ES. When a segment consists of many nodes, it will significantly increase the time overhead. In conclusion, adopting “parallel entanglement swapping between segments and sequential entanglement swapping within segments” is a rational approach when executing the tree-like model.

### 5.6. Time Synchronization and Node Swapping Failure Processing

**Figure 9:**
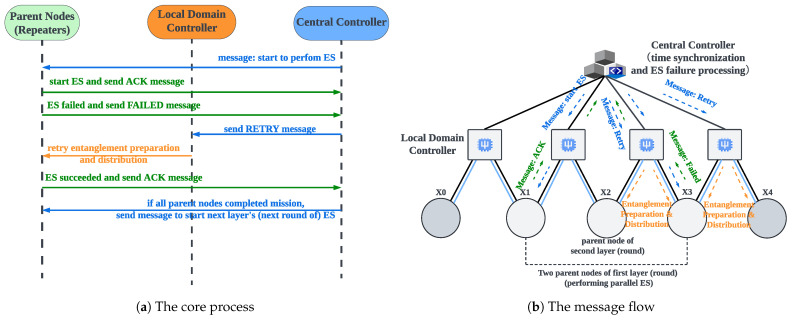
The time synchronization and on-demand retransmission mechanisms. (**a**) The vertically arranged lines represent the core procedural sequence of the time synchronization and on-demand retransmission mechanisms during a round of parallel ES. The blue, green, and orange lines represent the messages sent by the central controller, repeater, and local domain controller, respectively. (**b**) In the hierarchical quantum network architecture, messages are transmitted between devices via classical channels, as indicated by black solid lines. Local domain controllers prepare entanglements and distribute them via quantum channels, as indicated by light-blue solid lines.

The importance of implementing the time synchronization mechanism in parallel ES has been discussed in Section 4.2. Achieving time synchronization hinges on the presence of a control device that can coordinate the process of parallel ES. This control device must interact with repeaters and promptly acquire the outcomes of the ES operations. As illustrated in Figure 2, the central controller in the hierarchical architecture communicates with all repeaters via classical channels, thereby serving as the control device responsible for time synchronization in parallel ES. We developed and deployed a parallel ES control program, which includes a time synchronization function, in the central controller. Figure 9a,b show its core process and message flow, respectively. Before each round of parallel ES begins, the central controller sends instructions to all parent nodes in the current layer, as indicated by the first blue line in Figure 9a. Upon receiving the instruction, each repeater synchronously starts the ES and sends the first ACK message to the central controller, as indicated by the first green line in Figure 9a. If the ES operation is successful, each repeater sends the second ACK message to announce completion, as indicated by the third green line in Figure 9a. The central controller waits until it has received both ACK messages from all parent nodes in the current round. Once all responses have been collected, it confirms that the repeaters have completed ES successfully, and then proceeds to notify the parent nodes in the next layer to begin the following round of parallel ES, as indicated by the third blue line in Figure 9a. It is important to note that as the internal procedure of a composite parent node follows sequential ES, the central controller notifies the next repeater to begin ES only after the previous one has been completed. This coordination ensures the correct execution of sequential ES within the composite parent node.

As described in Section 4.3, ES failure is not rare, because environmental noise is pervasive. Therefore, ES failure processing is crucial for long-distance quantum communication. We propose the on-demand retransmission mechanism to process ES failure efficiently. The core idea of the on-demand retransmission mechanism is to utilize the central controller to identify nodes where ES has failed and promptly re-prepare and redistribute entanglements for those nodes. Compared to the traditional full-path retransmission mechanism, which re-prepares entanglements along the entire path, the on-demand approach targets only the failed nodes, thereby reducing latency and entanglement resource consumption in long-distance quantum communication. Figure 9a,b show the core process and message flow of the on-demand retransmission mechanism, respectively. As indicated by the second green line in Figure 9a, the repeater experiencing ES failure reports the event to the central controller via a FAILED message. The central controller immediately sends a RETRY message instructing the corresponding local domain controller to re-prepare and redistribute entanglements for the affected repeaters, as indicated by the second blue and first orange lines in Figure 9a. The repeater that encountered ES failure will immediately retry the ES operation upon receiving the new entanglement. We should emphasize that since the repeaters within a composite parent node (i.e., the repeaters belonging to the same segment) are pairwise adjacent and interdependent, and also perform sequential ES, when one repeater encounters an ES failure, both itself and its preceding repeaters should perform entanglement re-preparation and distribution. However, since repeaters belonging to different segments are non-adjacent and independent, and also perform parallel ES, the entanglement re-preparation and distribution triggered by ES failures in one segment do not affect the other segments, which reflects the concrete differences between the on-demand retransmission mechanism and the traditional full-path retransmission mechanism.

## 6. Parallel Segment Entanglement Swapping for Multi-User Concurrent Quantum Communication

### 6.1. Challenges of Resource Contention

In quantum network communication, once PSES has transformed a path into a tree-like model, all resources related to ES along this path, such as quantum channels, quantum memories, and quantum operation resources, will be locked until the communication is complete to ensure the successful execution of parallel ES. In point-to-point quantum communication, since there is only one path, that path exclusively accommodates the resources of each node. Thus, there is no resource contention. However, in multi-user concurrent quantum communication, resource contention may occur due to the simultaneous existence of multiple paths. For example, when multiple paths intersect at common nodes, the paths will compete for the resources of the common nodes. According to the resource locking mechanism of PSES, when a high-priority path locks the resources of a common node, other paths can only start performing ES after the communication of the high-priority path has been completed. We should note that this study assumes that the path priorities have been set. In fact, path priorities can be determined by the importance of user requests or other factors, but this is beyond the scope of this study. Moreover, we note that the multi-user quantum network in this paper is based on the hierarchical quantum network architecture [7]. Its physical scheme employs photons as communication qubits to transmit quantum information through optical fiber channels, while atoms, trapped ions, or superconducting circuits are used as the physical systems to implement data qubits (i.e., memory qubits) in repeaters and users. Although the different physical systems can affect the ES efficiency in multi-user quantum communication, this paper focuses on the ES strategy itself. Therefore, we assume that memory qubits across the network are realized using the same physical system. Additionally, in our simulation experiments, the variance caused by different physical systems can be effectively masked, so it does not impact the accuracy of the conclusions.

**Figure 10:**
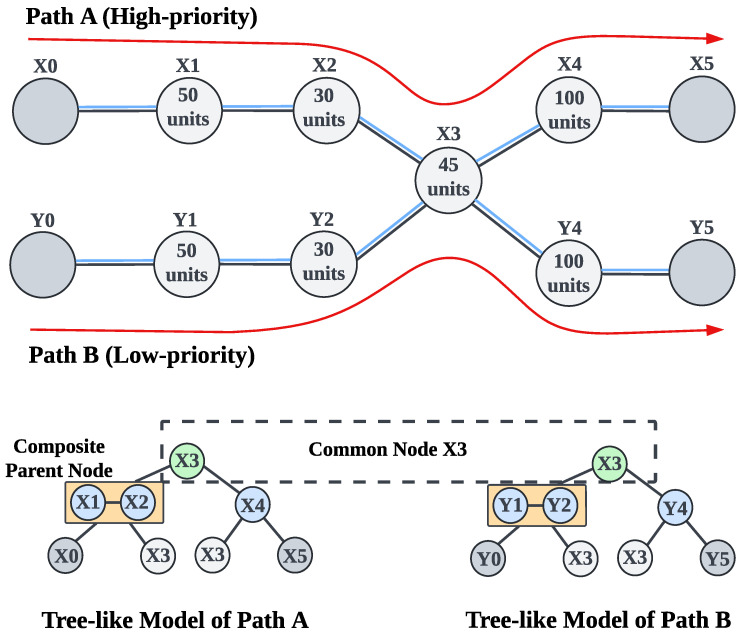
An example of resource contention in multi-user quantum communication. The high-priority Path A pre-empts the resource usage of the common node x3 before the low-priority Path B.

**Figure 11:**
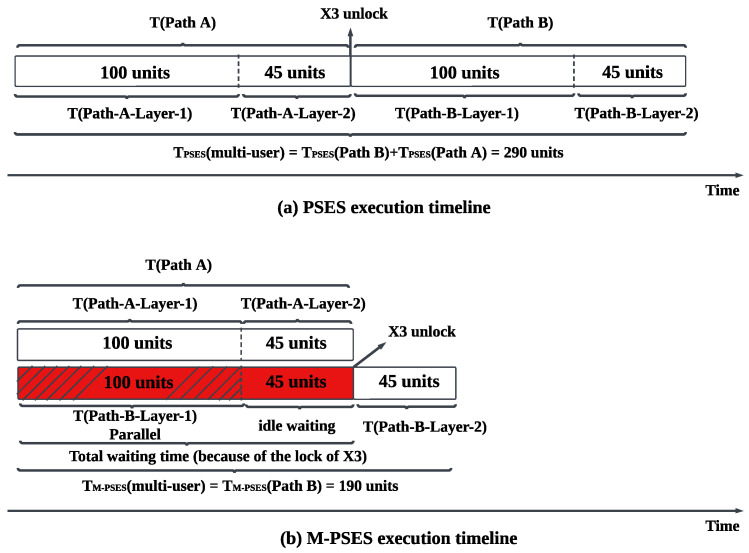
The execution timelines of PSES and M-PSES. (**a**) Due to the shortage of PSES, in the case of resource contention in multi-user concurrent quantum communication, the low-priority path can only perform ES after the high-priority path has completed the communication. (**b**) With the help of the resource locking mechanism and ES trigger signal mechanism of M-PSES, parallel ES can be performed between nodes belonging to the different paths, reducing time overhead.

We provide an example to illustrate the impact of resource contention on PSES. As shown in Figure 10, suppose there is a high-priority Path A and a low-priority Path B, with a common node x3 for both paths. For the sake of clarity in the example, we assume that the ES time cost for the corresponding nodes on both paths is identical. PSES transforms Path A and Path B into different tree-like models. According to the resource locking mechanism of PSES, since the two tree-like models share the common node x3, they cannot be executed in parallel. Based on the priority order, we can only execute the tree-like model of Path A first and then execute the tree-like model of Path B after the x3 resource has been released. Figure 11a shows the execution timeline for this example. We can see that PSES consumes 100 units of time to complete the ES for the first-layer nodes {x1, x2, x4} of Path A. It then consumes 45 units of time to complete the ES for the second-layer node {x3}, unlocking the resource of node x3. Afterward, it consumes 145 units of time to perform the ES for the first layer nodes {y1, y2, y4} and the second layer node {x3} of Path B. Therefore, for PSES, the total time required to complete ES for both paths is $T_{PSES}$(multi-user) = $T_{PSES}$(Path A) + $T_{PSES}$(Path B) = 290 units.

In conclusion, although PSES can perform parallel ES between nodes within a path to improve efficiency, it can not perform parallel ES between paths in the case of resource contention.

### 6.2. Design of Multi-User Parallel Segment Entanglement Swapping

To improve the efficiency of PSES in multi-user concurrent quantum communication, we propose Multi-user Parallel Segment Entanglement Swapping (M-PSES). The commonality between M-PSES and PSES is that they generate tree-like models in the same way (i.e., Layer Greedy or Segment Greedy), but the difference lies in their ES trigger signal mechanism and resource locking mechanism. Compared to PSES, the advantage of M-PSES is its ability to maximize parallel ES between nodes that belong to different paths. The core principles of M-PSES are as follows.

**Principle A.** The resources are released immediately after the node has completed ES, without waiting for the communication to finish.

**Principle B.** Two signals control the ES of a node. *Signal 1:* preceding nodes have completed the ES; *Signal 2:* the node resource is available.

**Principle C.** The ES of a node is started once the two signal conditions have been satisfied, without waiting for all node resources in the path to be locked.

Principle A explains the resource locking mechanism of M-PSES. The benefit of this mechanism is that it reduces the resource locking time in multi-user concurrent quantum communication, thereby decreasing the waiting time of low-priority paths. Since the node resources must be occupied until the node ES has been completed, the node resource locking time equals the sum of the ES time of the preceding nodes and the ES time required by the current node itself. For example, in the case shown in Figure 10, the resource locking time of the common node x3 is 145 units, because the ES time of the preceding nodes (i.e., x1, x2, and x4) is 100 units (i.e., the total ES time of Layer 1) and the ES time of x3 is 45 units. Additionally, if the common node is x2, the resource locking time will be reduced to 80 units, because the ES time of the preceding node (i.e., x1) is 50 units and the ES time of x2 is 30 units.

Principles B and C explain the ES trigger signal mechanism of M-PSES. The advantage of this mechanism is that it allows partial nodes in multiple paths to perform parallel ES during the waiting time, thereby increasing the efficiency of multi-user concurrent quantum communication. For example, in the case shown in Figure 10, all the nodes in Layer 1 of Path B do not rely on the ES result of the common node x3, so they can perform parallel ES with the nodes of Path A while waiting for x3 to be unlocked, thereby enhancing efficiency.

We intuitively demonstrate the advantages of M-PSES through the execution timeline. The execution timeline of M-PSES is shown in Figure 11b. The high-priority Path A is executed as usual. The low-priority Path B has a waiting time due to the resource of the common node x3 being locked by high-priority Path A, as shown by the red part in Figure 11b. During the waiting time, we can execute Layer 1 of Path B (i.e., {y1, y2, y4}), corresponding to the red shaded part. The red non-shaded part represents the idle waiting time. After the node x3 resource is unlocked, the ES for node x3 in Path B can be executed immediately because the preceding nodes in Path B (i.e., {y1, y2, y4}) have completed ES, and the two signal conditions are satisfied. Therefore, for M-PSES, the total time required to complete ES for both paths is $T_{\mathrm{M-PSES}}$(multi-user) = $T_{\mathrm{M-PSES}}$(Path B) = 190 units, which is significantly lower than the time cost of PSES. Indeed, three factors can affect the effectiveness of M-PSES: the number of common nodes, the number of paths, and the length difference between paths. We discuss these three situations in the following paragraphs.

The effectiveness of M-PSES can be calculated using Formula (6). In Formula (6), $E_{\mathrm{M-PSES}}$ represents the effectiveness of M-PSES. The numerator represents the time saved by M-PSES compared to PSES, and the denominator represents the time cost of PSES in multi-user concurrent quantum communication. Therefore, $E_{\mathrm{M-PSES}}$ reflects the efficiency with which M-PSES saves time. It is easy to observe that 0 <$T_{\mathrm{M-PSES}}$(multi-user) ≤$T_{PSES}$(multi-user), so we have 0 ≤$E_{\mathrm{M-PSES}}$< 1.

(6) $$\begin{array}{c} E_{\mathrm{M-PSES}}=\frac{T_{PSES}\left(\mathrm{multi-user}\right)-T_{\mathrm{M-PSES}}\left(\mathrm{multi-user}\right)}{T_{PSES}\left(\mathrm{multi-user}\right)} \end{array}$$

We need to discuss how the number of common nodes impacts the effectiveness of M-PSES. Firstly, when there are no common nodes between paths, both PSES and M-PSES enable all paths to perform ES in parallel, so $T_{\mathrm{M-PSES}}$(multi-user) = $T_{PSES}$(multi-user). Therefore, M-PSES cannot save time when there is no resource contention, and its effectiveness is 0. Secondly, when the number of common nodes between paths is 1∼N, M-PSES can save time, but its effectiveness decreases as the number of common nodes increases. The increase in the number of common nodes does not affect $T_{PSES}$(multi-user), but it increases the resource locking time in M-PSES, leading to longer waiting times, which, in turn, increases $T_{\mathrm{M-PSES}}$(multi-user) and reduces $E_{\mathrm{M-PSES}}$.

The number of paths also affects the effectiveness of M-PSES. As the number of paths increases, the number of nodes from different paths that can perform parallel ES during the waiting time also increases, enabling M-PSES to save more time. We can also observe this from the formula of $E_{\mathrm{M-PSES}}$. Formula (6) can be rewritten as $E_{\mathrm{M-PSES}}=1-\frac{T_{\mathrm{M-PSES}}\left(\mathrm{multi-user}\right)}{T_{PSES}\left(\mathrm{multi-user}\right)}$. As the number of paths grows, both $T_{PSES}$(multi-user) and $T_{\mathrm{M-PSES}}$(multi-user) exhibit an upward trend. However, the increase in $T_{\mathrm{M-PSES}}$(multi-user) is more gradual, as M-PSES effectively saves more time. Consequently, $E_{\mathrm{M-PSES}}$ exhibits a significant rise, reflecting its enhanced efficiency. For instance, in the case with two paths illustrated in Figure 10 and Figure 11, we can compute that $E_{\mathrm{M-PSES}}$ equals 0.34 according to Formula (6). When the scenario in Figure 10 and Figure 11 is extended to three paths, it becomes clear that $T_{PSES}$(multi-user) rises to 435 units. However, due to the increased number of nodes in Layer 1, which can execute parallel ES in the waiting time, $T_{\mathrm{M-PSES}}$ only increases to 235 units, leading to a corresponding increase in $E_{\mathrm{M-PSES}}$ to 0.46.

An increase in path length not only results in more nodes, but also leads to a longer ES time. In multi-user concurrent quantum communication, the length difference between paths can affect the effectiveness of M-PSES. Specifically, when the number of paths and common nodes is fixed, a minor length difference between paths means that more non-common nodes between paths can perform parallel ES, thereby allowing M-PSES to save time to a greater extent. Conversely, as the length difference between paths increases, there will be more non-common nodes in the long path that cannot be parallelized with nodes in the short path. This is because the long path is still running while the short path has completed its communication, causing the remaining nodes in the long path to lack matching nodes in the short path for parallel ES, thereby reducing the effectiveness of M-PSES. The aforementioned impact can also be observed from the formula of $E_{\mathrm{M-PSES}}$. Taking Figure 10 and Figure 11 as examples, under the baseline condition where both paths have the same length, the efficiency of M-PSES is given by $E_{\mathrm{M-PSES}(baseline)}=1-\frac{T_{\mathrm{M-PSES}}\left(\mathrm{multi-user}\right)}{T_{PSES}\left(\mathrm{multi-user}\right)}$. Suppose Path A increases in length (i.e., the length difference between Path A and B increases), resulting in an extra ES time of $T_{increase}$. Then, the efficiency of M-PSES is given by $E_{\mathrm{M-PSES}(increase)}=1-\frac{T_{\mathrm{M-PSES}}\left(\mathrm{multi-user}\right)+T_{increase}}{T_{PSES}\left(\mathrm{multi-user}\right)+T_{increase}}$. Since $T_{\mathrm{M-PSES}}\left(\mathrm{multi-user}\right)<T_{PSES}\left(\mathrm{multi-user}\right)$ when paths intersect, it is easy to see that $E_{\mathrm{M-PSES}(increase)}$ is smaller than $E_{\mathrm{M-PSES}(baseline)}$. In conclusion, as the length difference between paths increases, M-PSES becomes less efficient. In other words, M-PSES achieves maximum effectiveness when the lengths of paths are similar.

We also note that the entanglement preparation rate of quantum networks may affect the total ES time cost of a path. Although we assume a fixed entanglement preparation rate in Section 5.2, in practice, due to variations in devices and environments, the entanglement preparation rate is probabilistic and path-dependent. In other words, different paths may exhibit different entanglement preparation rates. This difference has a limited impact on PSES, but can significantly affect M-PSES. For PSES, since it forms a tree-like model for only a single path, differences in entanglement preparation rates across paths do not influence its performance. Furthermore, even if some nodes within a path experience reduced preparation rates due to environmental changes, PSES can mitigate this negative impact by adjusting the structure of the tree-like model. For M-PSES, since it constructs multiple tree-like models for several paths, differences in entanglement preparation rates across paths can affect its performance. Specifically, suppose the entanglement preparation rate of the high-priority path is reduced due to environmental changes. In that case, the performance of M-PSES can be significantly degraded, as the low-priority path may still need to wait for the resources of the high-priority path to be unlocked. However, suppose the entanglement preparation rate of the low-priority path decreases. In that case, its negative impact on M-PSES is relatively minor, since it does not block the high-priority path from performing ES.

### 6.3. Implementation of Multi-User Parallel Segment Entanglement Swapping

**Figure 12:**
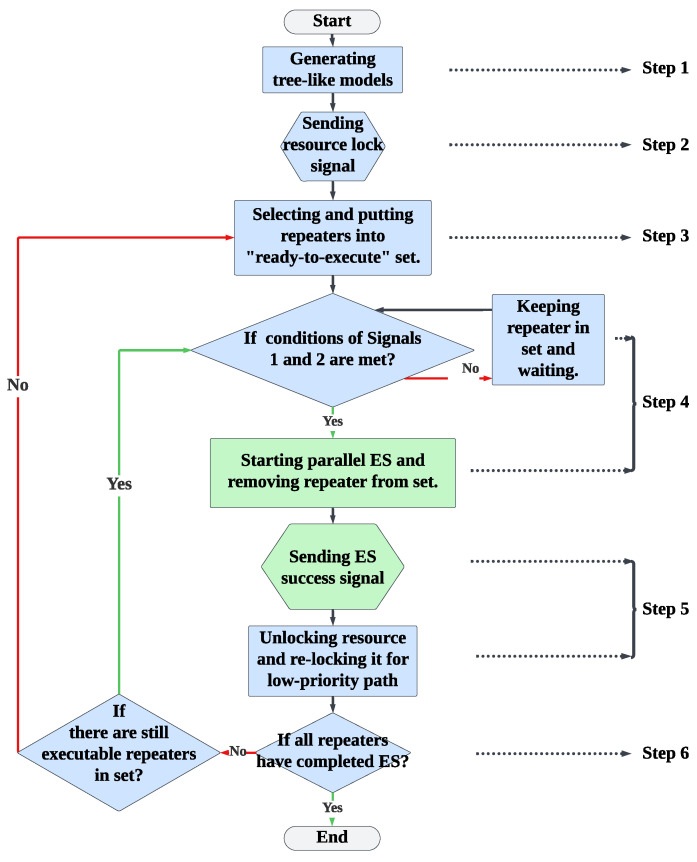
The execution process of M-PSES deployed in the central controller. Light-blue and light-green represent the operations performed by the central controller and repeaters, respectively. The hexagons represent the transmission of signals or information through the classical channel between the central controller and the repeaters.

The key to implementing M-PSES lies in controlling resource locking and transmitting *Signal 1* and *Signal 2*. The specific details of *Signals 1* and *2* have been provided in Principle B of Section 6.2. Notably, the central controller in the hierarchical architecture [7] shown in Figure 2 can serve as an ideal deployment platform for M-PSES. This is because the central controller can collect the information of all quantum repeaters in the network via classical channels and send instructions to them. Specifically, the M-PSES deployed in the central controller operates through a six-step process, as illustrated in Figure 12.

Step 1: M-PSES utilizes Layer Greedy or Segment Greedy to generate tree-like models for each path.

Step 2: The central controller requests each node to lock its resource according to path priority. During this process, if resource contention occurs at a common node, the resource will be locked by the high-priority path first, while the resource locking state of the common node in the low-priority path will be set to “pending”.

Step 3: The central controller selects the nodes for the first round of parallel ES based on the tree-like models, and adds them to a set called “ready-to-execute”.

Step 4: The central controller sends the instructions for starting ES to the nodes in the set that meet the conditions of *Signals 1* and *2*, and then removes them from the set. The remaining nodes that do not meet the conditions stay in the set and wait. During this step, the central controller continuously checks whether the remaining nodes in the set meet the conditions.

Step 5: When a node completes the operation, it sends a signal of ES completion to the central controller through the classical channel. The central controller records the information and immediately unlocks the resource of the node while locking the resource for the low-priority path.

Step 6: Once all the nodes that meet the conditions have completed ES, the central controller will return to Step 3, select the nodes for the next round of ES, and add them to the “ready-to-execute” set. The subsequent steps will then be repeated until the nodes in all paths have completed ES.

During the execution of M-PSES, the central controller performs the functions of signal transmission and node operation control. In terms of signal transmission, Steps 2 and 5 complete the transmission of resource locking and unlocking signals, as well as the node ES completion signals, thereby realizing Principles A and B of M-PSES. In terms of node operation control, Step 4 completes the recognition of *Signals 1* and *2*, thereby realizing Principle C of M-PSES. Therefore, the hierarchical quantum network architecture [7] plays a crucial role in deploying M-PSES and serves as the foundation for its implementation. We publish the source code of M-PSES in [33], facilitating future research.

In conclusion, M-PSES improves ES efficiency in multi-user concurrent quantum communication by mitigating the adverse effect of resource contention and maximizing parallel ES between nodes that belong to different paths. The core idea of M-PSES can provide three important inspirations for future research. First, mitigating resource contention in multi-user concurrent quantum communication will significantly optimize quantum communication efficiency. Second, other parallel ES strategies (e.g., IBT [24] and BBT [20,21,22,23]) can also leverage the hierarchical architecture to achieve parallel ES between nodes that belong to different paths, thereby enhancing ES efficiency in multi-user concurrent quantum communication. Third, the hierarchical architecture is not limited to parallel ES strategies; other quantum network protocols or strategies (e.g., entanglement routing [34], distribution [35], and purification [36]) that require node control can also leverage its control capabilities, as in the case of M-PSES.

## 7. Simulation and Evaluation

We used NetSquid [17] as the simulation platform, since it provides simulations of various key elements, including qubits, quantum repeaters, quantum memories, classical channels, quantum channels, and entanglement swapping. Another important reason for choosing NetSquid is that the hierarchical quantum network [7] is implemented on this platform. It provides a set of predefined hierarchical topologies, components, and communication protocol stacks, which greatly facilitate the simulation of PSES and M-PSES. The source code for PSES and M-PSES is available at [33].

### 7.1. Simulation Environment

**Figure 13:**
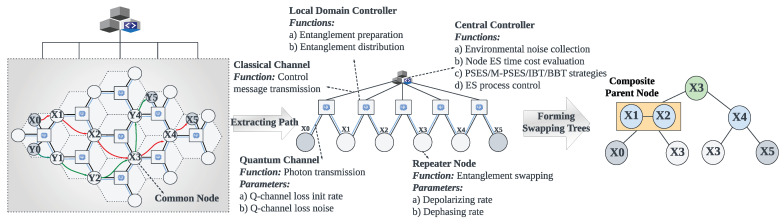
The simulation environment. A hierarchical quantum network with a cellular topology was established as the performance evaluation scenario. The parallel ES strategies are deployed in the central controller. The central controller can collect environmental noise to evaluate the node ES time cost. In point-to-point quantum communication, there is only one pair of users and a single path, such as the red path. In multi-user concurrent quantum communication, multiple paths may intersect, such as the red and green paths.

Figure 13 shows the simulation environment. We established a cellular topology of the hierarchical quantum network as the testing scenario for parallel ES strategies. In Figure 13, the central controller connects to each domain (regular hexagonal regions) via the classical channel, which is responsible for collecting environmental noise, evaluating node ES time costs, deploying parallel ES strategies (i.e., PSES, M-PSES, IBT, and BBT), and controlling the ES process (i.e., time synchronization and on-demand retransmission). Each domain may include the local domain controller, the quantum repeater, and possibly the quantum user. The local domain controller connects to quantum repeaters and users via quantum and classical channels, and is responsible for entanglement preparation and distribution. Repeaters are capable of performing quantum operations, and include two environmental noise parameters—the depolarizing rate and the dephasing rate. We note that three repeaters are sufficient to cover a domain in the cellular topology, as they can provide service to all adjacent hexagonal areas [7]. Classical channels are responsible for transmitting control information. Quantum channels are responsible for transmitting photons, and include two environmental noise parameters—the Q-channel loss init rate and Q-channel loss noise. Quantum users can serve as the source and destination of quantum network communication. In point-to-point quantum communication, there is only one pair of quantum users and a single path, such as the red path in Figure 13. In multi-user concurrent quantum communication, there are multiple pairs of quantum users and several paths, and these paths may intersect, such as the red and green paths in Figure 13. After the entanglement routing algorithm (e.g., Centralized Entanglement Routing [7]) confirms a path, a tree-like or binary tree model is formed by a parallel ES strategy and stored in the central controller. We should note that Figure 13 is a schematic diagram illustrating the simulation environment. The scale of the actual experimental topology was sufficient for us to increase both the length and number of paths for performance evaluation.

### 7.2. Methodology

We evaluated the performance of PSES, M-PSES, IBT, and BBT in point-to-point and multi-user concurrent quantum communication scenarios, respectively, to ensure a comprehensive analysis. The specific methodology was as follows.

**Point-to-point quantum communication scenarios.** We used the average ES time as the metric to evaluate the performance of PSES, M-PSES, IBT, and BBT under different numbers of hops and different standard deviations of node ES time cost. The more hops there are, the longer the path becomes. A higher standard deviation of node ES time cost indicates greater environmental noise difference among the nodes. The average ES time refers to the average time taken for the path to complete ES, which was obtained through repeated experiments. Obviously, a smaller average ES time indicates a higher generation rate of long-distance entanglement, implying better performance of strategies. Moreover, we compared the time consumption and entanglement consumption of on-demand retransmission and full-path retransmission. In our experiments, only generating long-distance entanglement with a fidelity greater than 0.5 was considered successful ES, because entanglement is deemed invalid when the fidelity is less than or equal to 0.5 [30]. We utilized the function provided by NetSquid [17] to obtain the fidelity of entanglement. The specific experimental designs were as follows.

(a) Average ES time vs. hops. We set the environmental noise parameters to fix the average node ES time cost at 1.4 and its standard deviation at 0.1. The performance of PSES, M-PSES, IBT, and BBT was evaluated as the number of hops increased from 6 to 10. For fairness, all strategies adopted the on-demand retransmission mechanism.

(b) Average ES time vs. standard deviation of node ES time cost. We fixed the number of hops and average node ES time cost at 6 and 1.4, respectively. Then, we explored the performance of PSES, M-PSES, IBT, and BBT as the standard deviation of node ES time cost increased from 0.1 to 0.5. It should be noted that an increase in the standard deviation of node ES time cost indicates a greater difference in environmental noise among nodes. For fairness, all strategies adopted the on-demand retransmission mechanism.

(c) On-demand retransmission vs. full-path retransmission. We compared the performance of the on-demand retransmission mechanism with the traditional full-path retransmission mechanism using a 5-hop path with an average node ES time cost of 1.4 and a standard deviation of 0.1. For fairness, we adopted PSES Layer Greedy as the common strategy for evaluating both mechanisms.

**Multi-user concurrent quantum communication scenarios.** We used the average ES time as the metric to evaluate the performance of PSES, M-PSES, IBT, and BBT under different numbers of common nodes, numbers of paths, and length differences between paths in multi-user concurrent quantum communication. It should be noted that in multi-user communication, the average ES time refers to the average time taken for all paths to complete ES. In particular, we introduce the effectiveness of M-PSES, denoted as $E_{\mathrm{M-PSES}}$ (see the Formula (6)), as an additional performance metric for M-PSES. This is because a higher $E_{\mathrm{M-PSES}}$ indicates that M-PSES can complete ES more quickly than PSES. For the sake of fairness, PSES, M-PSES, and IBT all employed the Layer Greedy algorithm, and all strategies adopted the on-demand retransmission mechanism. The specific experimental designs were as follows.

(d) Effectiveness of M-PSES vs. number of common nodes. We fixed the number of paths at 2 and set the length of each path to 5 hops. The performance of PSES, M-PSES, IBT, and BBT was evaluated as the number of common nodes increased from 0 to 4.

(e) Effectiveness of M-PSES vs. number of paths. We fixed the number of common nodes at 1 and set the length of each path to 5 hops. Then, we explored the performance of PSES, M-PSES, IBT, and BBT as the number of paths increased from 2 to 6.

(f) Effectiveness of M-PSES vs. length difference between paths. We fixed the number of common nodes at 1 and the number of paths at 2, initially setting the length of both paths to 5 hops (i.e., the length difference between paths was 0). Then, we increased the length of one path to enlarge the length difference between paths, and observed the performance of PSES, M-PSES, IBT, and BBT.

### 7.3. Performance in Point-to-Point Quantum Communication Scenarios

**Figure 14:**
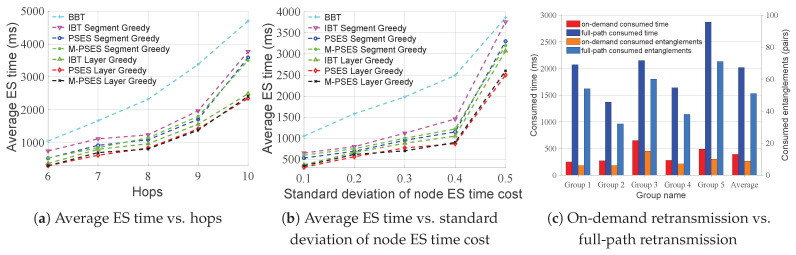
The performance of parallel ES strategies in point-to-point quantum communication scenarios. In Subfigure (**a**), the average node ES time cost is fixed at 1.4, and its standard deviation is fixed at 0.1. In Subfigure (**b**), the average node ES time cost is fixed at 1.4, and the path consists of 6 hops. In Subfigure (**c**), the average node ES time cost is fixed at 1.4, its standard deviation is fixed at 0.1, and the path consists of 5 hops.

**Average ES time vs. hops.** The experimental results are shown in Figure 14a. We can see that the average ES time of all strategies increases as the number of hops increases, because a longer path requires more repeaters to perform ES, leading to higher time consumption. From the cyan line, it can be observed that BBT consistently has the highest average ES time across different numbers of hops, as it does not consider the environmental noise of nodes. The purple, blue, and green lines show that the average ES times of PSES Segment Greedy and M-PSES Segment Greedy are close to each other and lower than that of IBT Segment Greedy. This is because both PSES and M-PSES adopt the tree-like model, allowing them to save more time compared to IBT. However, due to the absence of resource contention in point-to-point quantum communication, M-PSES can not fully leverage its advantages, resulting in similar performance between M-PSES and PSES. The leaf-green, red, and black lines show that the average ES times of PSES Layer Greedy and M-PSES Layer Greedy are close to each other and lower than that of IBT Layer Greedy. The reason for this phenomenon is the same as above. However, from the perspective of algorithms, we find that strategies based on Layer Greedy are always better than those based on Segment Greedy. This is because Layer Greedy can obtain the optimal solution for each layer, while Segment Greedy can only obtain the optimal solution for each segment, resulting in a performance gap. In conclusion, the performance ranking of the different strategies is PSES Layer Greedy ≈ M-PSES Layer Greedy > IBT Layer Greedy > PSES Segment Greedy ≈ M-PSES Segment Greedy > IBT Segment Greedy > BBT. Moreover, we observe that under the same algorithm (i.e., Segment Greedy or Layer Greedy), the difference in the average ES time between PSES and IBT does not increase as the number of hops increasse, indicating that the number of hops does not impact their performance gap.

**Average ES time vs. standard deviation of node ES time cost.** The experimental results are shown in Figure 14b. We can see that under different standard deviations of node ES time cost, the performance ranking remains consistent with the previous experiment, which is PSES Layer Greedy ≈ M-PSES Layer Greedy > IBT Layer Greedy > PSES Segment Greedy ≈ M-PSES Segment Greedy > IBT Segment Greedy > BBT. However, unlike the previous experiment, when comparing the differences between the purple and blue lines or the red and leaf-green lines at standard deviations of node ES time cost from 0.1 to 0.5, it is evident that under the same algorithm (e.g., Segment Greedy or Layer Greedy), the gap in average ES time between PSES and IBT increases as the standard deviation of node ES time cost increases, indicating that the greater the variation in environmental noise among nodes, the more pronounced the advantages of PSES become. This phenomenon occurs because a greater difference in environmental noise leads to a greater variation in node ES time costs, providing PSES with more opportunities to generate composite parent nodes, thereby saving more time.

**On-demand retransmission vs. full-path retransmission.** The difference between the on-demand retransmission mechanism and the traditional full-path retransmission mechanism is that the former performs entanglement re-preparation only for the nodes related to the ES failure, whereas the latter performs entanglement re-preparation for all nodes. The experimental results are shown in Figure 14c. We can see that in all five groups, the full-path retransmission mechanism results in higher time consumption and entanglement consumption compared to the on-demand retransmission mechanism. On average, the time consumption and entanglement consumption for the on-demand retransmission mechanism are only 390 ms and 8.8 pairs, respectively, while for the full-path retransmission mechanism, they are 2020 ms and 51 pairs, respectively. Because the on-demand retransmission mechanism can accurately identify and handle nodes with ES failures, it can save approximately 80% of the time cost and 80% of the entanglement consumption compared to the traditional method, thereby effectively improving the ES efficiency.

### 7.4. Performance in Multi-User Concurrent Quantum Communication Scenarios

**Figure 15:**
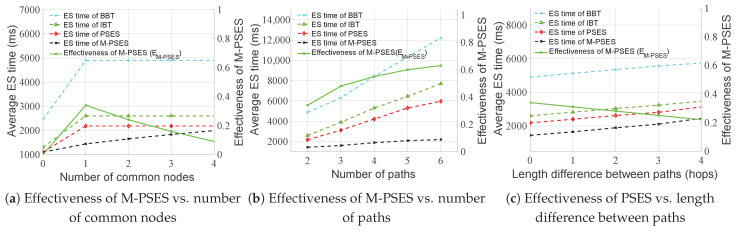
The performance of parallel ES strategies in multi-user concurrent quantum communication scenarios. In Subfigure (**a**), the number of paths is fixed at 2, and the length of both paths is fixed at 5 hops. In Subfigure (**b**), the number of common nodes is fixed at 1, and the length of all paths is fixed at 5 hops. In Subfigure (**c**), the number of common nodes is fixed at 1, and the number of paths is fixed at 2; the length of both paths is initially set to 5 hops (i.e., the length difference between paths is 0 hops); and the hops of one path are then increased to enlarge the length difference.

**Effectiveness of M-PSES vs. number of common nodes.** The experimental results are shown in Figure 15a. The dashed lines show that M-PSES performs better than PSES, IBT, and BBT in the multi-user concurrent quantum communication scenarios, because it can leverage the ES trigger signal and resource locking mechanisms to mitigate resource contention. First, we can observe that when there are no common nodes between paths, the average ES time of all strategies remains low, with M-PSES and PSES showing the same ES time consumption, indicating that the effectiveness of M-PSES is 0. The reason for this phenomenon is that when the paths do not intersect and experience no resource contention, all strategies can perform parallel ES between paths. Second, we can observe that when the number of common nodes ranges from 1 to 4, the average ES time of PSES, IBT, and BBT remains constant, but is higher compared to the scenario with no common nodes. In contrast, the average ES time of M-PSES shows a gradual upward trend, although it is always lower than that of PSES, IBT, and BBT. Additionally, the green solid line shows that when paths have common nodes, the effectiveness of M-PSES decreases as the number of common nodes increases. The reason for this phenomenon is that when paths have common nodes and resource contention occurs, the resource locking mechanism in PSES, IBT, and BBT forces sequential ES between paths to be performed. In contrast, M-PSES allows parallel ES to be performed between the partial nodes, which have no resource contention and belong to different paths, thereby reducing the average ES time. However, as the number of common nodes increases, the number of nodes with no resource contention decreases, which, in turn, causes the average ES time of M-PSES to rise and its effectiveness to decrease. Therefore, we can conclude that if paths have common nodes in multi-user concurrent quantum communication, M-PSES can effectively save ES time and improve the quantum communication efficiency. Moreover, the fewer the number of common nodes, the higher the effectiveness of M-PSES.

**Effectiveness of M-PSES vs. number of paths.** The experimental results are shown in Figure 15b. First, we can observe that as the number of paths increases, the average ES time of all strategies increases, but the performance of M-PSES remains the best. The reason for this phenomenon is that as the number of paths increases, the competition for common nodes becomes more intense, leading to longer waiting times for paths, which, in turn, results in an increase in the overall time overhead. Second, we can see that as the number of paths increases, the effectiveness of M-PSES gradually increases, meaning that the increase in the average ES time for M-PSES is smaller than that for PSES. The reason for this phenomenon is that as the number of paths increases, although the waiting time for paths also increases, M-PSES can utilize this waiting time to allow more nodes, which have no resource contention and belong to different paths, to perform parallel ES, thereby saving more time. Therefore, we can conclude that when the number of common nodes remains constant, and the more paths there are, the higher the effectiveness of M-PSES and the more ES time it can save.

**Effectiveness of PSES vs. length difference between paths.** The experimental results are shown in Figure 15c. First, we can observe that as the length difference between paths increases, the average ES time of all strategies shows an upward trend, but the performance of M-PSES remains the best. The reason for this phenomenon is that when the length of one path increases (i.e., the length difference between paths increases), the additional nodes introduce extra ES time costs. For PSES, IBT, and BBT, since parallel ES between nodes belonging to different paths is unavailable, the additional ES time cost naturally adds up to the original time cost. For M-PSES, as the length difference between paths increases, the additional nodes in the long path are less likely to find matching nodes in the short path for parallel ES, resulting in the extra ES time cost adding to the original time cost. Second, we can observe that as the length difference between paths increases, the efficiency of M-PSES decreases. This phenomenon occurs because a larger length difference between paths results in more non-common nodes in the long path being unable to find matching nodes in the short path for parallel ES, which decreases the effectiveness of M-PSES.

### 7.5. Discussion

First, we discuss the performance of each strategy in point-to-point quantum communication scenarios. Under the same algorithm (i.e., Segment Greedy or Layer Greedy), the performance of PSES and M-PSES is superior to that of BBT and IBT. This is because both PSES and M-PSES adopt the tree-like model, which introduces the composite parent node, fully utilizing idle waiting time to allow more nodes to perform ES simultaneously. Additionally, the performance of PSES and M-PSES is similar in point-to-point quantum communication scenarios, as no resource contention occurs, preventing M-PSES from leveraging its advantages. Moreover, we can see that the greater the difference in environmental noise among nodes, the more pronounced the performance advantages of PSES and M-PSES compared to IBT and BBT. Furthermore, compared to the traditional full-path retransmission mechanism, the on-demand retransmission mechanism can reduce both the time and the entanglement consumption by 80%. From an algorithmic perspective, combining the algorithm-level experiments in Section 5.4.3, we can conclude that in point-to-point quantum communication scenarios, when the number of path hops is less than or equal to seven (i.e., short path), PSES Layer Greedy is the best choice of strategy, as it offers the best performance while keeping the computational time at the microsecond level (i.e., <1 ms). When the number of path hops exceeds seven (i.e., long path), the computational time of PSES Layer Greedy increases rapidly and approaches milliseconds; among the strategies that maintain computational time at the microsecond level, PSES Segment Greedy performs the best, making it the optimal choice of strategy. In summary, in point-to-point quantum communication scenarios, PSES and M-PSES perform better than other strategies. However, due to the absence of resource contention, the advantages of M-PSES can not be fully leveraged.

Second, we discuss the performance of M-PSES in multi-user concurrent quantum communication scenarios. When resource contention occurs, with the help of ES trigger signal and resource locking mechanisms, M-PSES can effectively utilize the resource waiting time to perform parallel ES between paths as much as possible. Therefore, M-PSES can save more time than other strategies and enhance the efficiency of multi-user concurrent quantum communication. Additionally, we note that M-PSES is more suitable for deployment in compact topologies (e.g., star and cellular topologies) rather than sparse topologies (e.g., ring grid topologies). Specifically, in compact topologies, multiple paths are more likely to intersect with common nodes, providing more opportunities for M-PSES to be effective. In contrast, in sparse topologies, there are more alternative paths, making it less likely for paths to intersect, leaving limited opportunities for M-PSES to be effective. Referring to classical networks, future quantum networks will inevitably involve multi-user concurrent communication scenarios. M-PSES can be effectively applied in such scenarios, making it practically significant. In fact, the core idea of M-PSES can also inspire other parallel ES strategies (e.g., IBT [24] and BBT [20,21,22,23]) to address resource contention problems in multi-user concurrent quantum communication.

The upcoming third-generation quantum repeater [37,38] may adopt Tree Cluster State encoding [39] instead of relying on long-distance entanglement. The more encoded qubits there are in the Tree Cluster States, the greater the fault tolerance of the quantum information [40]. However, increasing the number of encoded qubits also leads to higher resource consumption and lower transmission efficiency. In fact, fewer encoded qubits can be allocated to paths with lower environmental noise to reduce resource consumption, as long as we can collect the environmental noise of nodes. Inspired by this study, an interesting direction for exploration is how to leverage the global perspective of hierarchical architecture [7] to optimize the allocation of encoded qubits in Tree Cluster States across different paths.

## 8. Conclusions

This study focuses on the efficiency of ES strategies in quantum network communication, with a particular emphasis on multi-user concurrent quantum communication scenarios. Because multi-user concurrent quantum communication consists of multiple point-to-point quantum communications, we first investigate existing ES strategies in point-to-point quantum communication scenarios and identify their challenges. To address these challenges, we propose an efficient strategy, PSES, for point-to-point quantum communication scenarios. Compared to other strategies, PSES reduces the time cost of ES by introducing the tree-like model, and realizes time synchronization and on-demand retransmission mechanisms by utilizing a hierarchical quantum network architecture. Furthermore, we propose M-PSES, which can mitigate resource contention in multi-user concurrent quantum communication by utilizing the ES trigger signal and resource locking mechanisms. M-PSES performs better than other strategies in multi-user concurrent quantum communication scenarios. Although we are still a long way from a practical quantum network, the significance of this study lies in providing a possible ES solution and inspiring future research into ES strategies.

Future work can be divided into three parts: (1) PSES and M-PSES belong to the network layer, using the advantages of hierarchical architecture to improve the efficiency of ES, which may inspire researchers to develop more great protocols in different layers based on these advantages. (2) PSES and M-PSES can be further developed, such as by introducing entanglement purification [41] and quantum error correction [42], to further improve the generation rate of long-distance entanglement. (3) Inspired by PSES and M-PSES, it is worth considering utilizing the advantage of hierarchical architecture to realize Tree Cluster State encoding [39] in third-generation repeaters, rather than the ES strategy in first-generation or second-generation repeaters.

## Figures and Tables

**Table 1:** Comparison of algorithms.

	Heuristic Method		Optimization Method
Algorithm	Layer Greedy	Segment Greedy	Dynamic Programming [24]
Principle	Find the optimal layer solution	Find the optimal segment solution	Find the optimal tree solution
Problem Size (Granularity)	Middle (Layer level)	Small (Segment level)	Big (Tree level)
Performance	Middle (see Figure 6 and Figure 7)	Low (see Figure 6 and Figure 7)	High [24]
Time Cost	Middle (see Figure 8)	Low (see Figure 8)	Huge [24]

**Table 2:** Best-fit algorithms and strategies for different scenarios.

	Short-Path Scenarios (Hops ≤ 7)	Long-Path Scenarios (Hops > 7)
Best-fit scheme	PSES Layer Greedy	PSES Segment Greedy
Reason	Best performance (Figure 6 and Figure 7); μs-level algorithm time cost (Figure 8)	Acceptable performance (Figure 6 and Figure 7); μs-level algorithm time cost, regardless of hops (Figure 8)

## Data Availability

The data are available in a publicly accessible repository. The data presented in this study are openly available in reference [33].
